# Karyopherin **α** deficiency contributes to human preimplantation embryo arrest

**DOI:** 10.1172/JCI159951

**Published:** 2023-01-17

**Authors:** Wenjing Wang, Yoichi Miyamoto, Biaobang Chen, Juanzi Shi, Feiyang Diao, Wei Zheng, Qun Li, Lan Yu, Lin Li, Yao Xu, Ling Wu, Xiaoyan Mao, Jing Fu, Bin Li, Zheng Yan, Rong Shi, Xia Xue, Jian Mu, Zhihua Zhang, Tianyu Wu, Lin Zhao, Weijie Wang, Zhou Zhou, Jie Dong, Qiaoli Li, Li Jin, Lin He, Xiaoxi Sun, Ge Lin, Yanping Kuang, Lei Wang, Qing Sang

**Affiliations:** 1 Institute of Pediatrics, Children’s Hospital of Fudan University, State Key Laboratory of Genetic Engineering, Institutes of Biomedical Sciences, Shanghai Key Laboratory of Medical Epigenetics, Fudan University, Shanghai, China.; 2 Laboratory of Nuclear Transport Dynamics, National Institutes of Biomedical Innovation, Health and Nutrition, Osaka, Japan.; 3 NHC Key Lab of Reproduction Regulation, Shanghai Institute for Biomedical and Pharmaceutical Technologies, Shanghai, China.; 4 Reproductive Medicine Center, Northwest Women’s and Children’s Hospital, Xi’an, China.; 5 Reproductive Medicine Center, Jiangsu Province Hospital, Jiangsu, China.; 6 Clinical Research Center for Reproduction and Genetics in Hunan Province, Reproductive and Genetic Hospital of CITIC-Xiangya, Changsha, China.; 7 Reproductive Medicine Center, Henan Provincial People’s Hospital, Zhengzhou, China.; 8 Key Laboratory of Human Reproduction and Genetics, Department of Reproductive Medicine, Nanchang Reproductive Hospital, Nanchang, China.; 9 Shanghai Key Laboratory of Maternal Fetal Medicine, Clinical and Translational Research Center, Shanghai First Maternity and Infant Hospital, School of Medicine, Tongji University, Shanghai, China.; 10 Reproductive Medicine Center, Shanghai Ninth Hospital, Shanghai Jiao Tong University, Shanghai, China.; 11 Shanghai Ji’ai Genetics and IVF Institute, Obstetrics and Gynecology Hospital, and; 12 State Key Laboratory of Genetic Engineering and Collaborative Innovation Center for Genetics and Development, School of Life Sciences, Fudan University, Shanghai, China.; 13 Bio-X Center, Key Laboratory for the Genetics of Developmental and Neuropsychiatric Disorders, Ministry of Education, Shanghai Jiao Tong University, Shanghai, China.

**Keywords:** Genetics, Reproductive Biology, Fertility, Genetic diseases, Population genetics

## Abstract

Preimplantation embryo arrest (PREMBA) is a common cause of female infertility and recurrent failure of assisted reproductive technology. However, the genetic basis of PREMBA is largely unrevealed. Here, using whole-exome sequencing data from 606 women experiencing PREMBA compared with 2,813 controls, we performed a population and gene–based burden test and identified a candidate gene, karyopherin subunit *α*7 (*KPNA7*). In vitro studies showed that identified sequence variants reduced KPNA7 protein levels, impaired KPNA7 capacity for binding to its substrate ribosomal L1 domain-containing protein 1 (RSL1D1), and affected KPNA7 nuclear transport activity. Comparison between humans and mice suggested that mouse KPNA2, rather than mouse KPNA7, acts as an essential karyopherin in embryonic development. *Kpna2^–/–^* female mice showed embryo arrest due to zygotic genome activation defects, recapitulating the phenotype of human PREMBA. In addition, female mice with an oocyte-specific knockout of *Rsl1d1* recapitulated the phenotype of *Kpna2^–/–^* mice, demonstrating the vital role of substrate RSL1D1. Finally, complementary RNA (cRNA) microinjection of human *KPNA7*, but not mouse *Kpna7*, was able to rescue the embryo arrest phenotype in *Kpna2^–/–^* mice, suggesting mouse KPNA2 might be a homologue of human KPNA7. Our findings uncovered a mechanistic understanding for the pathogenesis of PREMBA, which acts by impairing nuclear protein transport, and provide a diagnostic marker for PREMBA patients.

## Graphical abstract

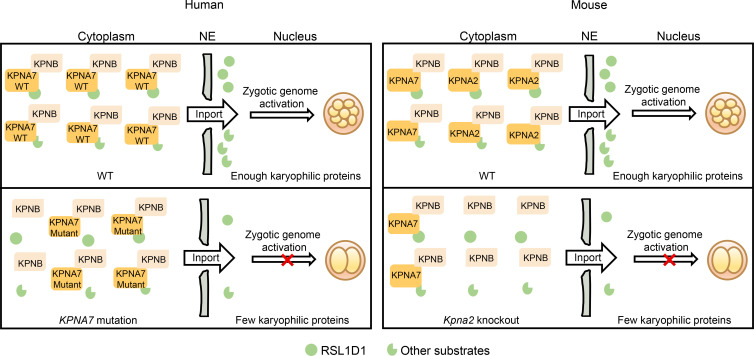

## Introduction

Successful human reproduction requires normal embryonic development. Preimplantation embryo arrest (PREMBA) is a common reason for recurrent failure of in vitro fertilization (IVF) or intracytoplasmic sperm injection (ICSI) and leads to female infertility (1). If all human embryos generated by IVF or ICSI are taken into consideration, about 10% of them are arrested at the primary cleavage stages (2). For PREMBA patients, morphologically normal eggs can be retrieved and fertilized and they can undergo the first division, but most of the embryos are arrested before the 8-cell stage on day 3 or before the blastocyst stage on day 5, resulting in no viable embryos for implantation. Currently, the underlying genetic determinants and molecular mechanisms behind human PREMBA are largely unrevealed.

A few genetic factors responsible for human PREMBA have been identified, most of which encode subcortical maternal complex (SCMC) proteins such as *PADI6* (3), *NLRP2* (4), *NLRP5* (4), and *KHDC3L* (5). This is consistent with the observation that abnormal function of SCMC proteins results in PREMBA (6). Until now, there have existed few reports on other pathways or molecules contributing to human PREMBA. In addition, the reported PREMBA-relevant genes were conventionally discovered by cosegregation analysis in pedigrees consisting of 2 or more patients or by homozygosity mapping in consanguineous families (3, 4). Such approaches can only explain a small percentage of pedigrees or consanguineous patients, highlighting the limited knowledge of the genetic basis for the remaining nonconsanguineous patients with the sporadic form of PREMBA. Until now, there has been no genetic burden analysis based on whole-exome sequencing (WES) of a large cohort of patients with the sporadic form of PREMBA. Gene-based burden tests that calculate accumulated associations built on multiple variants of each gene are a powerful strategy for identifying novel candidate genes in various disorders (7, 8). We therefore recruited a large cohort of PREMBA patients and performed a gene-based burden test by comparing sequencing data of PREMBA patients with our in-house control database.

Our genetic burden analysis revealed a burden signal for karyopherin subunit α7 (*KPNA7*) in the patient group under a recessive model. *KPNA7* encodes the karyopherin α7 protein (also referred to as importin α8) that mediates the transport of proteins between the nucleus and cytoplasm (9). The causal relationship between *KPNA7* variants and human PREMBA was confirmed by a series of in vitro and in vivo studies. We also showed that there is a species difference in *KPNA7* function between humans and mice by comparing corresponding karyopherin-member KO mice. *Kpna2^–/–^* mice, but not *Kpna7^–/–^* mice, could recapitulate the PREMBA phenotype in patients with *KPNA7* variants. In addition, female mice with an oocyte-specific KO of ribosomal L1 domain-containing protein 1 (*Rsl1d1*^OO–/–^) recapitulated the phenotype of *Kpna2* KO, demonstrating the vital role of maternal RSL1D1. Microinjection of human *KPNA7* rather than mouse *Kpna7* could rescue the phenotype of embryo arrest resulting from mouse *Kpna2* KO, suggesting mouse KPNA2 (mKPNA2) might be the homologue of human KPNA7 (hKPNA7).

## Results

### Genetic burden analysis identified candidate gene KPNA7.

**Figure 1:**
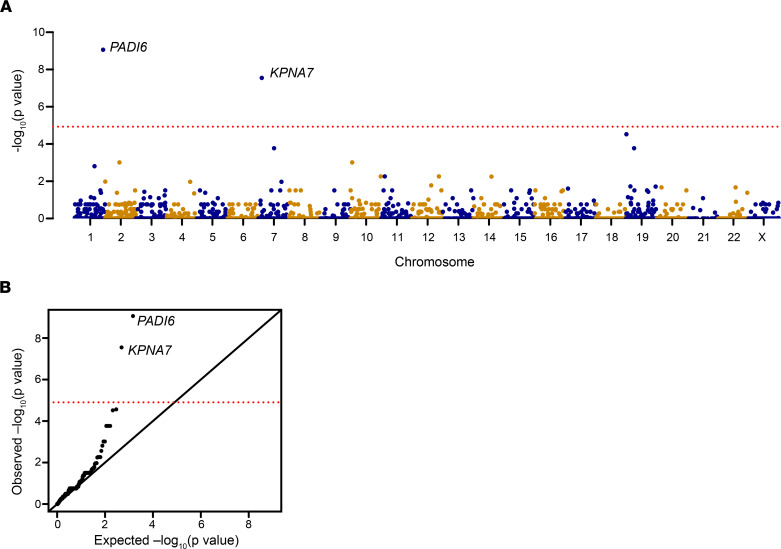
Burden testing shown as a Manhattan plot and a QQ plot. Two genes, including the known *PADI6*, showed significant enrichment of rare variants in patients. (**A**) Manhattan plot of –log_10_ (*P* value) from a cohort of PREMBA patients. (**B**) QQ plot of the –log_10_ (*P* value). The *x* axis represents the expected –log_10_ (*P* value) under the uniform distribution of *P* values. The *y* axis shows the observed –log_10_ (*P* value) from the burden testing data. The red dotted line shows the Bonferroni’s corrected significance threshold *P* = 1.25 × 10^–5^ (0.05/3993).

To discover genes responsible for human PREMBA in patients with the sporadic form, we performed WES in 606 PREMBA patients and 2,813 healthy controls. Variant calling and quality control were performed jointly across the samples (Supplemental Figure 1; supplemental material available online with this article; https://doi.org/10.1172/JCI159951DS1). Following the phasing of individual-level genotype data, 49,274 putative functional variants across 3,993 genes were identified, and a standard collapsing analysis under a gene-based burden test for recessive diplotypes was performed. As expected, we observed a significant enrichment of biallelic rare variants in the well-studied gene *PADI6* (Genbank NM_207421.4) in 12 patients but not in controls (*P* = 8.78 × 10^−10^) (Figure 1, A and B). The positive signal for *PADI6* demonstrates the feasibility of our sample size and analysis system.

In addition to *PADI6*, another strong genetic burden signal pointed to *KPNA7* (Figure 1, A and B), a member of the karyopherin α family of genes. Rare variants in *KPNA7* (NM_001145715.3) occurred in 10 patients and none in controls (*P* = 2.88 × 10^−8^), which surpassed the Bonferroni’s corrected significance threshold (0.05/3993, *P* = 1.25 × 10^−5^).

**Figure 2:**
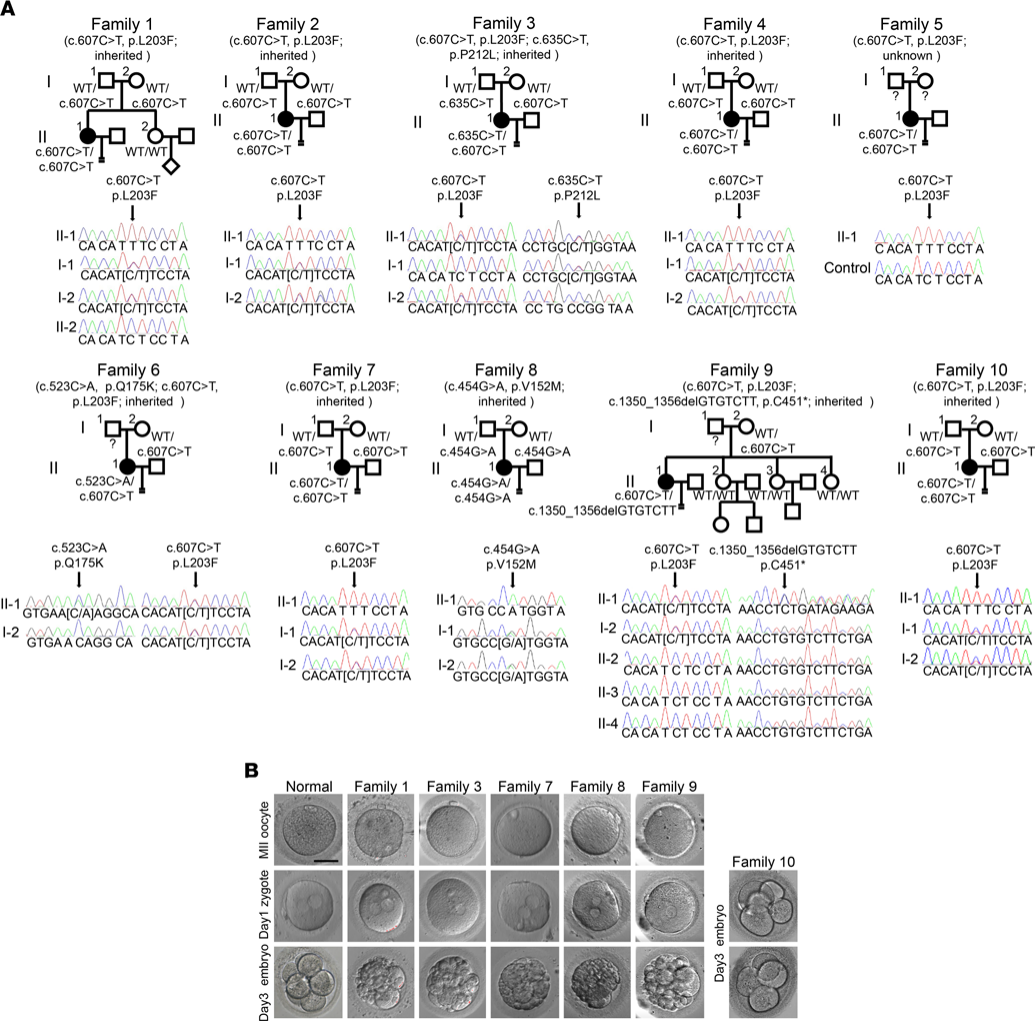
*KPNA7* biallelic variants identified in 10 independent families. (**A**) Pedigrees of the 10 families affected by infertility with Sanger sequencing confirmation below. Squares denote male family members, circles denote female members, solid circles denote affected individuals, and equals signs represent infertility. (**B**) Phenotypes of oocytes and early stage embryos from 5 of the patients. Scale bar: 80 μm.

To validate the genetic evidence for *KPNA7*, Sanger sequencing was used to confirm the variants and inheritance pattern (Figure 2A). The patients in families 1, 2, 4, 5, 7, and 10 all carried a homozygous recurrent variant (c.C607T, p.L203F), while the patients in families 3, 6, and 9 had compound heterozygous variants, including the recurrent variant (c.C607T, p.L203F), combined with c.C635T, p.P212L, c.C523A, p.Q175K, and c.1350_1356delGTGTCTT,p.C451*, respectively. There were no homozygotes for the recurrent variant either in public databases or our in-house control databases. Homozygosity mapping analysis suggested a low probability of founder effect for the recurrent variant L203F (Supplemental Figure 2). The patient from family 8 had a homozygous missense variant (c.G454A, p.V152M). The variants in families 1, 2, 3, 4, 7, 8, and 10 followed an obvious recessive inheritance pattern, while one of the alleles was of maternal origin in families 6 and 9, thus excluding the possibility that the compound heterozygous variants came from the same allele. The inheritance pattern in family 5 was uncertain due to the lack of parental samples. Overall, 4 missense and 1 nonsense variant were identified in *KPNA7* from 10 patients. *KPNA7* is localized in chromosome 7, and the predicted protein consists of an N-terminal importin β binding domain and 10 armadillo (ARM) repeats (10) (Supplemental Figure 3A). Residues affected by missense variants were located between the third and fourth ARM repeats and were highly conserved among different vertebrate species (Supplemental Figure 3A). In silico analysis predicted that the 4 missense variants were functionally damaging or possibly damaging (Supplemental Table 1). In addition, we found that *KPNA7* was highly and specifically expressed in human oocytes and early embryos, but was nearly undetectable in most somatic tissues (Supplemental Figure 3B), suggesting its potentially important roles during early embryonic development. The population-based genetic burden analysis implicated a candidate gene, *KPNA7*, leading to PREMBA.

### Clinical features.

All PREMBA patients with *KPNA7* variants had regular menstrual cycles and normal sex hormone levels, but had suffered from infertility for many years. Several rounds of IVF/ICSI cycles had been attempted, but all ended in failure. Detailed clinical information is summarized in Supplemental Table 2 and Figure 2B. Briefly, for the patient in family 1, the majority of the cleaved embryos were arrested at the 2- to 6-cell stage on day 3 and were discarded after failing to reach the blastocyst stage. Only a few 6- to 9-cell embryos were frozen and were implanted, but no pregnancy was established. For the patients in family 2 and family 3, most of the fertilized oocytes underwent normal cleavage, but failed to reach the blastocyst stage after extended culture or failed to establish pregnancy. The patient in family 4 had limited embryos that underwent normal cleavage, but all were arrested at the 3- to 5-cell stage. The patient in family 5 had a few viable embryos on day 3, but they failed to form blastocysts on day 5. The patients in families 6, 7, and 8 had limited viable embryos and blastocysts, and they all failed to establish pregnancy. For the patients in family 9 and family 10, embryos could undergo cleavage, but no embryos were viable on day 3 or they failed to form blastocysts after culture.

In summary, in most of the patients carrying biallelic *KPNA7* variants, normal PB1 oocytes could be retrieved and varying numbers of zygotes could be obtained. However, after the first rounds of cleavage, embryos were arrested or could not establish pregnancy after implantation, thus showing the phenotype of PREMBA.

### Pathogenic variants in KPNA7 disrupted its binding ability with its substrate and showed reduced nuclear transport activity.

**Figure 3:**
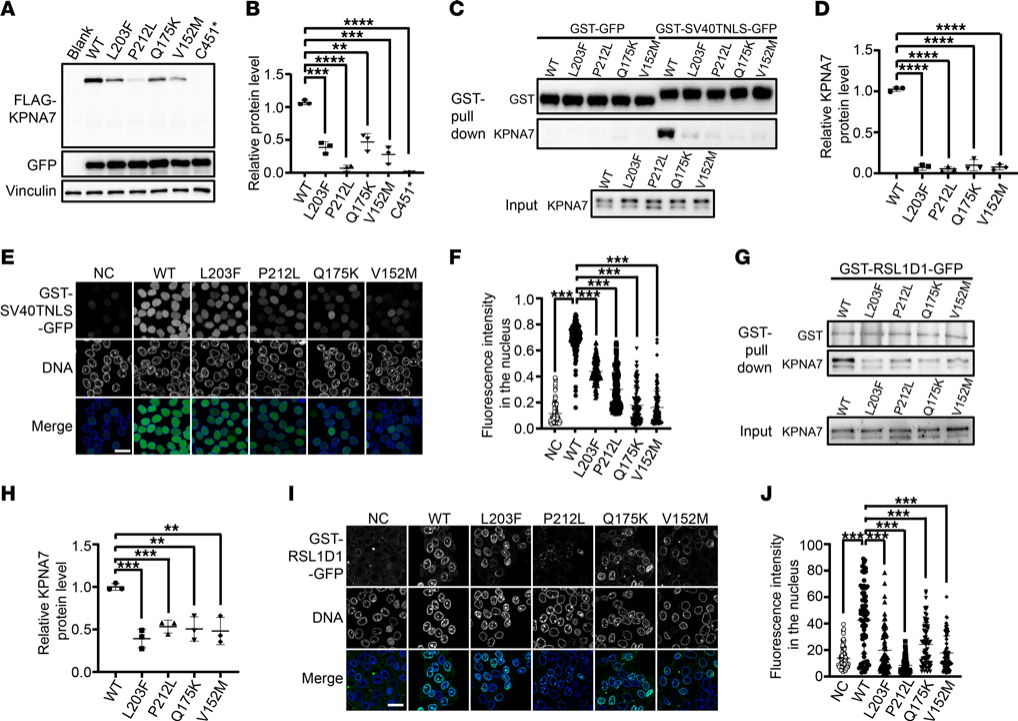
Effects of *KPNA7* variants in vitro. (**A**) Immunoblot of WT and the 5 mutant KPNA7 proteins in HEK293T cells. (**B**) Relative protein levels of KPNA7. Relative amounts of KPNA7 were calculated after normalizing to the endogenous vinculin and the exogenous GFP protein level. (**C**) Pull-down assay using purified GST-GFP/GST-SV40TNLS-GFP and WT or missense mutant KPNA7 with GST beads. Missense alterations affect the ability of KPNA7 to bind to substrate SV40TNLS. (**D**) Quantitative analysis of the KPNA7 protein levels in **C**. Relative amounts of KPNA7 were calculated after normalizing to the GST-SV40TNLS-GFP protein level. (**E**) In vitro nuclear transport assays of purified WT and missense mutant KPNA7 with the transport substrate GST-SV40TNLS-GFP. Missense alterations affect transport ability of KPNA7 to its substrates SV40TNLS. Negative control (NC) means no addition of the KPNA7 protein. DNA was stained with DAPI. Scale bar: 30 μm. (**F**) Quantitative analysis of KPNA7 transport capacity in **E**. A total of 100 cells were counted. (**G**) Pull-down assay using purified GST-RSL1D1-GFP and WT or missense mutant KPNA7 with GST beads. (**H**) Quantitative analysis of KPNA7 protein levels in **G**. Relative amounts of KPNA7 were calculated after normalizing to the GST-RSL1D1-GFP protein level. (**I**) In vitro nuclear transport assays with the transport substrate GST-RSL1D1-GFP. Missense alterations affect transport ability of KPNA7 to RSL1D1. DNA was stained with DAPI. Scale bar: 30 μm. (**J**) Quantitative analysis of KPNA7 transport capacity in **I**. Seventy cells were counted. All quantitative data in Figure 3 are represented as individual values with mean ± SD. *n* = 3 biological replicates. One-way ANOVA. **P < 0.01; ***P < 0.001; ****P < 0.0001.

To assess the impact of identified variants on KPNA7 function in vitro, FLAG-tagged vectors with WT or mutant *KPNA7* were transfected into HEK293T cells to measure protein levels by Western blotting, and GFP-tagged vectors were cotransfected to evaluate transfection efficiency. As a result, the protein levels of all missense mutant KPNA7 (p.L203F, p.P212L, p.Q175K, p.V152M) were significantly lower than WT, while p.C451* mutant KPNA7 was completely undetectable (Figure 3, A and B). Meanwhile, immunofluorescent assays were performed in transfected HeLa cells to detect the subcellular distribution. As shown in Supplemental Figure 4, although the nuclear localization of KPNA7 was not affected in the mutant groups, the signal intensity was very faint and was visible only under high exposure settings, which was consistent with the Western blotting result in HEK293T cells.

It has been reported that importin α binds its substrates to exert nuclear transport functions through the major classical nuclear localization signal (cNLS) binding pocket that resides between the second and fourth ARM repeats (10). KPNA7 can bind and transport SV40TNLS-containing proteins (11). In view of the amino acid alignment showing that all 4 patient-derived missense variants were located within the second and fourth ARM domains (Supplemental Figure 3A), we speculated that the binding ability of KPNA7 with SV40TNLS proteins may be disrupted by these variants. WT and missense mutant KPNA7 proteins were purified for pull-down assays together with glutathione *S*-transferase–tagged (GST-tagged) SV40TNLS-GFP. As shown in Figure 3, C and D, the interaction between KPNA7 and SV40TNLS-GFP was strongly disrupted by the missense variants. Furthermore, in vitro nuclear transport assays showed that mutant KPNA7 showed significantly reduced SV40TNLS protein transport activity compared with WT (Figure 3, E and F). Besides, it is known that the GTP fixed form of Ran (Q69LRanGTP) inhibits nuclear import by importin β (12). We next tested transport capacity of KPNA7 under exposure to Q69LRanGTP. Transport abilities of mutant KPNA7 proteins were more sensitive to Q69LRanGTP than WT (Supplemental Figure 5, A–C), further confirming the destructive effects of the *KPNA7* variants. Thus, the physiological function of KPNA7 was significantly impaired by the patient-derived pathogenic variants.

### RSL1D1 is a downstream substrate for KPNA7.

Although a few potential cargos of KPNA7 have been identified in pancreatic cancer cell lines and KPNA7-overexpressing HEK293 T lines (11, 13), up until now, there has, to our knowledge, been no report on specific candidate substrates that function in human oocytes and early embryos. Thus, we set the following two selection criteria. First, using the NCBI’s Blastp database, we looked for human proteins that contained nuclear localization sequences that are highly similar to SV40TNLS (PKKKRKV). By sequence alignment, 26 proteins were identified (Supplemental Table 3). Secondly, mRNA expression of candidate substrates should be high in human oocytes or early embryos according to our in-house RNA-Seq results using human oocytes or early embryos at different stages. Finally, RSL1D1, with the nuclear localization sequence PKKPKV and high expression in oocytes, was identified as one of the likely candidate substrates for KPNA7. RSL1D1, cellular senescence-inhibited gene (CSIG), is implicated in regulating cell cycle and cell senescence (14). GST pull-down assays and in vitro transport assays further supported the substrate property of RSL1D1 toward KPNA7 (Figure 3, G and I, and Supplemental Figure 5D). Next, to explore the influence of patient-derived variants on the interaction between KPNA7 and RSL1D1, GST-RSL1D1-GFP pull-down and immunoprecipitation experiments were carried out. As indicated in Figure 3, G and H, and Supplemental Figure 5D, all missense variants significantly reduced the ability of KPNA7 to bind to RSL1D1. Furthermore, the nuclear import of RSL1D1 was also decreased by the KPNA7 mutants (Figure 3, I and J). In summary, RSL1D1 is a downstream substrate of KPNA7, and the pathogenic variants impaired the interaction between KPNA7 and RSL1D1.

### mKPNA2 may be equivalent to hKPNA7 and acted as the key karyopherin α in mouse embryos.

**Figure 4:**
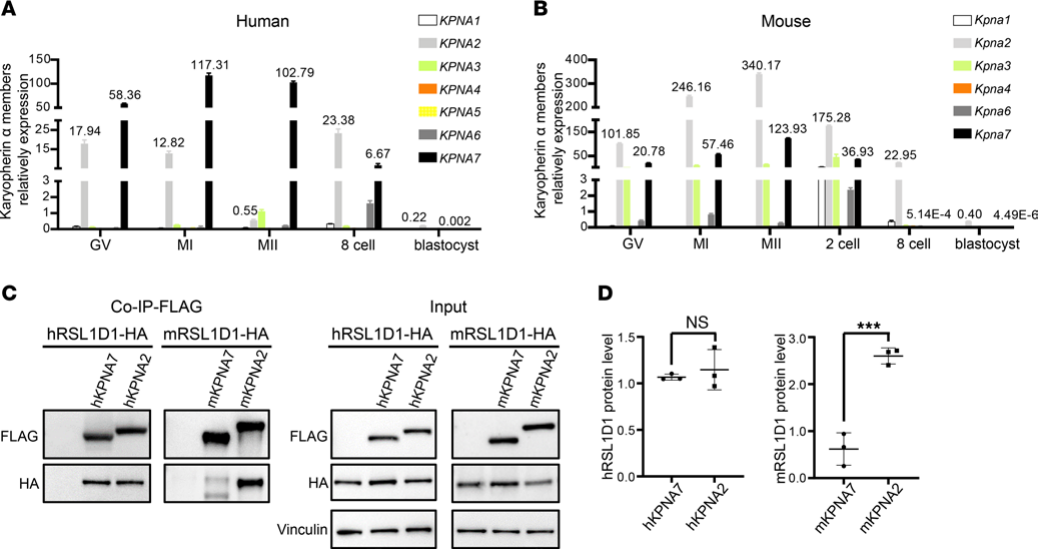
Different expression patterns and binding affinity for RSL1D1 between KPNA7 and KPNA2 in humans and mice. (**A**) Comparison of karyopherin α gene expression patterns in humans by qRT-PCR. (**B**) Comparison of karyopherin α expression patterns in mice by qRT-PCR. *KPNA7* was the most highly expressed subtype in human oocytes, while *KPNA2* was the most highly expressed subtype in mouse oocytes. Values of *KPNA2* and *KPNA7* are indicated above bar graphs. (**C**) Immunoprecipitation from HEK293T cells expressing FLAG-tagged WT KPNA7 or KPNA2 and HA-tagged RSL1D1 with anti-FLAG beads. (**D**) Quantification of **C**. Data are represented as individual values with mean ± SD. *n* = 3 biological replicates. Unpaired 2-sided *t* test. ***P < 0.001.

In order to recapitulate the phenotype of embryo arrest in mice, we first generated *Kpna7^L222F^* knockin mice (corresponding to human recurrent variant L203F) (Supplemental Figure 6A). However, all homozygous *Kpna7*^L222F^ female mice were healthy and fertile (Supplemental Figure 6, B and C). We compared the expression of WT mouse KPNA7 (mKPNA7) and that of the corresponding human mutants in transfected cells. Mouse *Kpna7* corresponding variants had no effect on mKPNA7 protein levels (Supplemental Figure 6D), while human *KPNA7* variants remarkably reduced the protein levels of hKPNA7 (Figure 3A). This suggests different effects of the same variants on hKPNA7 and mKPNA7. To further mimic the embryo arrest phenotype in mice, we constructed *Kpna7*-KO (*Kpna7^–/–^*) mice (Supplemental Figure 6, A, E, and F). Unexpectedly, homozygous *Kpna7^–/–^* female mice with both C57BL/6 and ICR backgrounds were also healthy and fertile (Supplemental Figure 6, G and H). We therefore speculated that there could exist functional compensation or redundancy for *KPNA7* in mice. Given that there exist 7 and 6 subtypes of karyopherin α in humans and mice, respectively (15), we compared the expression of different subtypes in oocytes and early embryos in both humans and mice by quantitative real-time PCR (qRT-PCR) (Figure 4, A and B). Strikingly, these subtypes showed expression patterns that differed in human and mouse oocytes. In humans, *KPNA7* showed the highest expressed karyopherin α, while in mice, *Kpna2* showed the highest expressed karyopherin α compared with *Kpna7* and other members of the karyopherin α family (Figure 4, A and B). Moreover, considering the fact that KPNA2 and KPNA7 are classified into the same subfamily of karyopherin α proteins (10), we inferred that in mice, KPNA2, rather than KPNA7, might be the homologue of hKPNA7.

In addition, we compared the binding capacity of hKPNA7 and mKPNA2 with RSL1D1, respectively. We found that the interaction of hKPNA7 and human KPNA2 (hKPNA2) with human RSL1D1 (hRSL1D1) was similar (Figure 4, C and D). However, mKPNA2 showed dramatically stronger interaction with mouse RSL1D1 (mRSL1D1) than mKPNA7 (Figure 4, C and D), implying that mKPNA2, instead of mKPNA7, plays an essential role in mouse early embryos and functions through the same downstream target RSL1D1 as hKPNA7.

### mKPNA2 depletion recapitulated the phenotype resulting from hKPNA7 deficiency by affecting the nuclear transport of RSL1D1.

**Figure 5:**
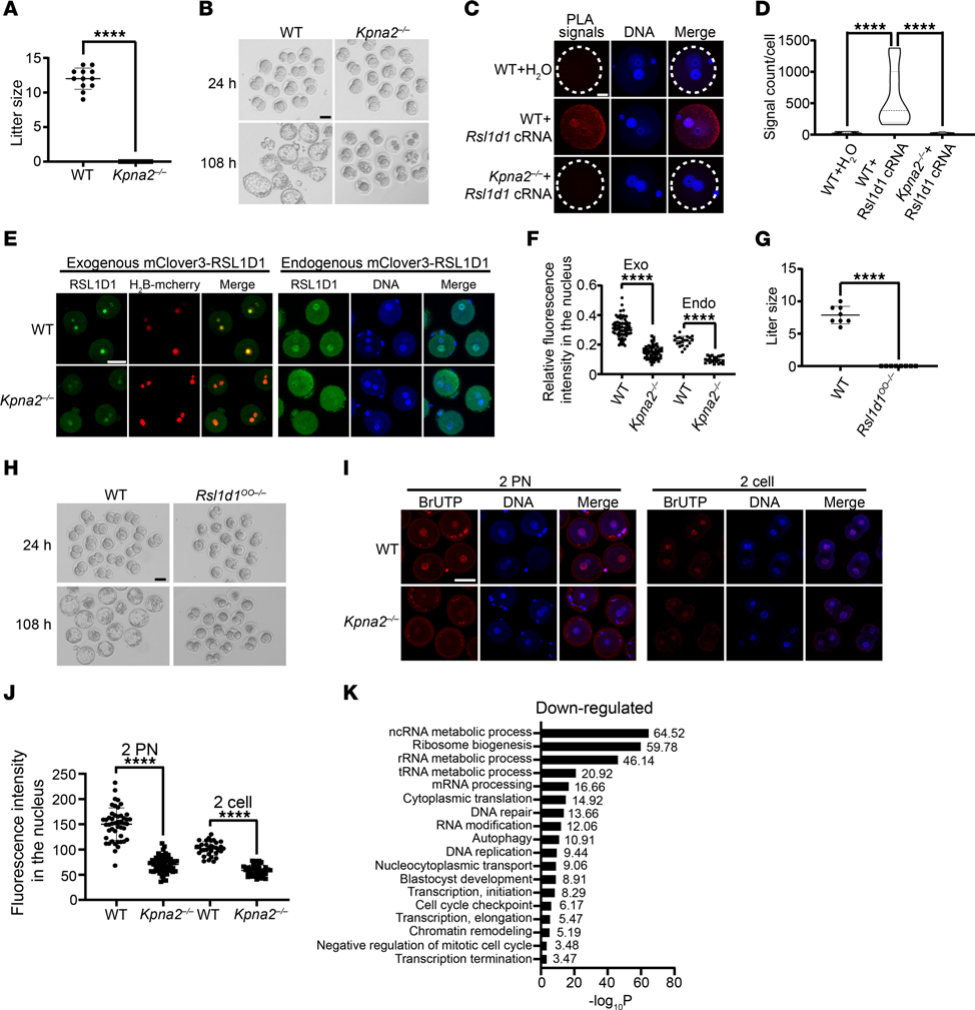
Infertility of *Kpna2*^–/–^ females. (**A**) Statistical analysis of the reproductive ability of *Kpna2^–/–^* mice. *n* = 12. Data are represented as individual values with mean ± SD. (**B**) Two-cell stage embryos and blastocysts derived from WT and *Kpna2^–/–^* females at 24 hours and 108 hours after IVF. Scale bar: 50 μm. (**C**) PLA-detecting interaction between exogenous RSL1D1-HA and endogenous KPNA2. Scale bar: 20 μm. (**D**) Quantitative analysis of PLA signals in **C**. Violin plots are shown with median as well as lower (25%) and upper (75%) quartiles. Ten cells in 3 biological replicates were counted. One-way ANOVA. (**E**) Localization of exogenous mClover3-RSL1D1 and endogenous RSL1D1 in zygotes from WT and *Kpna2^–/–^* females. H_2_B-mcherry was exogenously injected as the marker of the chromosomes. Scale bar: 50 μm. (**F**) Relative fluorescence intensity of RSL1D1 in the nucleus compared with the total cell in zygotes shown in **E**. Exo, exogenous; Endo, endogenous. Data are represented as individual values with mean ± SD. *n* = 3 biological replicates. Unpaired 2-sided *t* test. (**G**) Statistical analysis of the reproductive ability of *Rsl1d1*^OO–/–^ mice. *n* = 8. Data are represented as individual values with mean ± SD. (**H**) Phenotype of embryos from *Rsl1d1*^OO–/–^ females at 24 hours and 108 hours after IVF. Scale bar: 50 μm. (**I**) Incorporation of BrUTP, which marks the synthesis of nascent transcripts in 2 PN zygotes and 2-cell embryos from WT and *Kpna2^–/–^* females. Scale bar: 50 μm. (**J**) Fluorescence intensity of BrUTP in **I**. Data are represented as individual values with mean ± SD. *n* = 3 biological replicates. Unpaired 2-sided *t* test. (**K**) Gene Ontology analysis of downregulated genes after maternal KO of *Kpna2* in 2-cell embryos. ***P < 0.001; ****P < 0.0001.

In view of the possibility that m*KPNA2*, rather than m*KPNA7*, plays a role equivalent to that of h*KPNA7*, we next constructed *Kpna2*-KO mice (*Kpna2^–/–^*) (Supplemental Figure 7, A–C). *Kpna2^–/–^* females on a C57BL/6 background were difficult to obtain, and the few homozygotes that were obtained showed small body size and small ovaries. Only a few oocytes were super ovulated, but all were arrested at the 2-cell stage after fertilization. It has been reported that mouse models for one gene may produce varying degrees of phenotype on different genetic backgrounds (16). Thus, we attempted to produce ICR background *Kpna2^–/–^* mice. Unlike C57BL/6 background mice, *Kpna2^–/–^* mice on an ICR background were easy to obtain and exhibited normal body and ovary size. Mating *Kpna2^–/–^* female mice with WT males yielded no offspring (Figure 5A), while *Kpna2^+/–^* females and *Kpna2^–/–^* males were fertile. Oocytes from ICR *Kpna*2*^–/–^* females could be fertilized normally, and most zygotes could complete the first cleavage (Figure 5B and Supplemental Table 4). However, extended in vitro culture revealed embryo arrest at the 2-cell stage. Only a small number of embryos developed into the 4-cell stage at 72 hours after fertilization, but none of them progressed to the morula stage (Figure 5B and Supplemental Table 4), thus mimicking the phenotype of PREMBA resulting from KPNA7 deficiency in human embryos. Thus, in humans, KPNA7 plays an essential role during embryonic development, while the function of KPNA7 in mice is replaced by that of KPNA2, suggesting that the *Kpna2^–/–^* mouse is a reasonable animal model for elucidating the pathogenesis of PREMBA resulting from *KPNA7* dysfunction.

We have demonstrated that mKPNA2 functions via the same downstream substrate RSL1D1 as hKPNA7 (Figure 4C). Further proximity ligation assay (PLA) validates the interaction between mRSL1D1 and mKPNA2 (Figure 5, C and D). Next, to determine the effect of *Kpna2* depletion on the nuclear localization of RSL1D1 in mice, we monitored the entry of RSL1D1 into the nucleus by microinjecting complementary RNA (cRNA) of mClover3-tagged mouse *Rsl1d1* into zygotes together with H_2_B-mcherry as the marker of chromosomes and by immunofluorescence of endogenous RSL1D1. All zygotes from the WT group showed strong nuclear localization of RSL1D1, while in the *Kpna2^–/–^* group, the RSL1D1 nuclear signals were extremely weak and sporadic (Figure 5, E and F). In contrast, the entry of RSL1D1 into the nucleus was not affected in mouse *Kpna7^–/–^* zygotes (Supplemental Figure 7D), indicating that mKPNA2, instead of mKPNA7, plays an important role in RSL1D1 nuclear import.

To evaluate the role of RSL1D1 during embryonic development, we first downregulated *Rsl1d1* expression in mouse 2 pronuclei (PN) zygotes in vitro. Compared with the control group, almost all *Rsl1d1* knockdown embryos were arrested at the morula stage, and *Rsl1d1* cRNA injection was able to rescue the phenotype (Supplemental Figure 7, E and F), demonstrating that the embryo arrest phenotype resulted from *Rsl1d1* knockdown. To evaluate the role of *Rsl1d1* on embryonic development in vivo, we generated mice with an oocyte-specific KO of *Rsl1d1* on a C57BL/6 background (*Rsl1d1*^OO–/–^) (Supplemental Figure 7, G and H). *Rsl1d1*^OO–/–^ homozygous females were completely infertile (Figure 5G). To further evaluate the phenotype, we performed IVF of *Rsl1d1*^OO–/–^ females with WT males. Most oocytes (about 81.6%) from *Rsl1d1*^OO–/–^ females could be fertilized normally, and 64.5% of zygotes could develop to the 2-cell stage (Figure 5H and Supplemental Table 5). However, few of these 2-cell embryos were able to progress to the 4-cell stage (Figure 5H and Supplemental Table 5), exactly recapitulating the phenotype of *Kpna2^–/–^* female mice. These results further implied that hKPNA7 and mKPNA2 exert their function through the specific downstream substrate RSL1D1 during embryonic development.

### Kpna2^–/–^ embryos showed ZGA defects that partially contributed to embryo arrest.

It is well known that zygotic genome activation (ZGA) is essential for normal embryonic development (17, 18), and a group of nuclear localization factors needs to be imported into the nucleus so that these factors can perform their roles during ZGA (19). Considering the fact that *Kpna2^–/–^* zygotes showed reduced nuclear localization of RSL1D1 (Figure 5, E and F), we hypothesized that nuclear transport of nuclear factors is impaired and that ZGA events in *Kpna2^–/–^* preimplantation embryos may be impaired. It has been reported that minor ZGA is initiated as early as 5 hours after pronucleus formation and that major ZGA is initiated at the 2-cell stage and is essential for embryo development beyond the 2-cell stage (20, 21). Therefore, we monitored the transcription of endogenous genes in 1-cell and 2-cell embryos using 5-bromouridine 5′-triphosphate (BrUTP) incorporation. WT embryos showed normal transcription, while the signal in *Kpna2^–/–^* embryos was significantly reduced (Figure 5, I and J), suggesting that the ZGA events may be severely disturbed by *Kpna2* depletion. As a control, the ZGA process in *Kpna7^–/–^* zygotes was not affected (Supplemental Figure 7I), which also suggested that mKPNA2 might be the homologue of hKPNA7.

To further elucidate the main affected pathways responsible for embryo arrest upon *Kpna2* depletion, we performed RNA-Seq using in vitro–fertilized 2-cell embryos from ovarian-stimulated *Kpna2^–/–^* and *Kpna2^+/–^* mice. According to Gene Ontology analysis, 5,907 genes were downregulated in a variety of cellular processes, including RNA metabolism, RNA modification, transcription, and cytoplasmic translation, etc. (Figure 5K). qRT-PCR showed the decreased expression of transcription- and translation-related genes, especially genes related to the commonly known ZGA marker RNA polymerase II (22) (Supplemental Figure 7J), indicating that the phenotype of embryo arrest in *Kpna2^–/–^* mice at least partially resulted from ZGA defects. In light of the similar role between hKPNA7 and mKPNA2, this mechanism may also explain the PREMBA phenotype in patients with KPNA7 pathogenic variants.

### Human KPNA7 cRNA supplementation can overcome embryo arrest in Kpna2^–/–^ mice.

**Figure 6:**
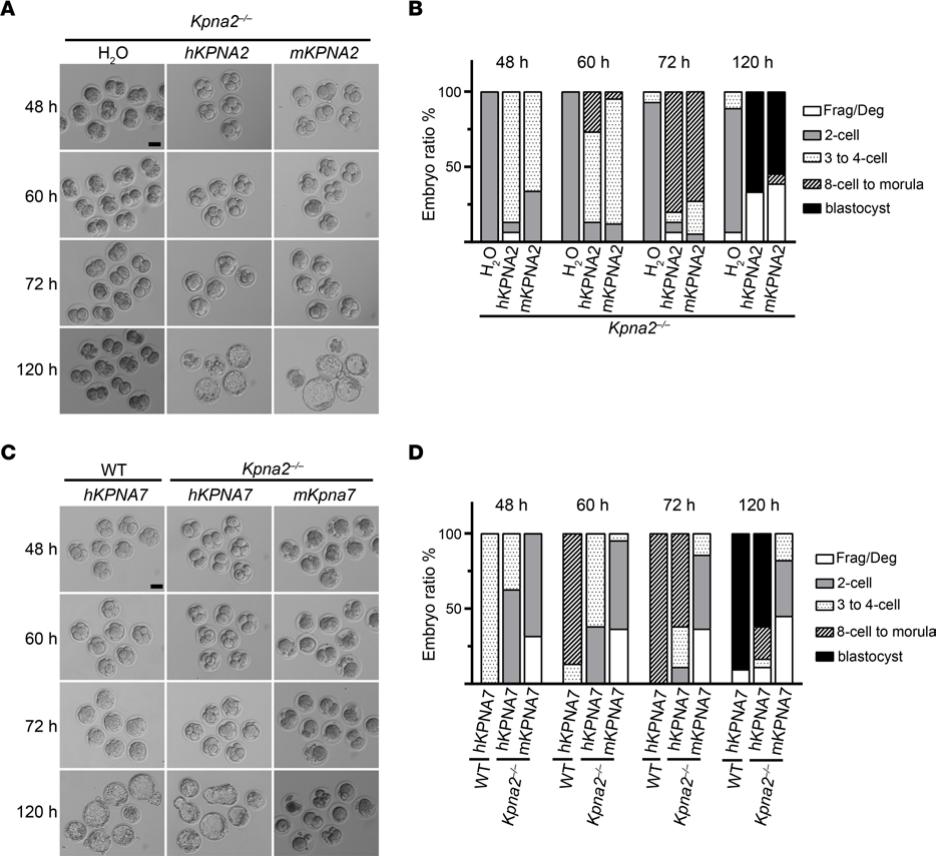
Phenotypic rescue by cRNA injection. (**A**) Phenotypic rescue by cRNA injection of human and mKPNA2 with concentration of 1,000 ng/μL. Scale bar: 50 μm. (**B**) Statistical analysis of **A**. Data are shown as means. *n* = 3 biological replicates. Frag/Deg, fragmented or degenerated. (**C**) Phenotypic rescue by cRNA injection of human and mouse *KPNA7* with concentration of 3,000 ng/μL. h*KPNA7*, rather than m*Kpna7*, was able to rescue the phenotype of PREMBA. Scale bar: 50 μm. (**D**) Statistical analysis of **C**. Data are represented as means. *n* = 3 biological replicates.

To explore a potential therapeutic strategy for PREMBA patients resulting from KPNA7 deficiency, we performed rescue experiments in *Kpna2^–/–^* mice through microinjection of *KPNA2* or *KPNA7* cRNA. First, to evaluate the rescue efficiency at the zygote stage, we microinjected cRNAs of human and mouse *KPNA2* into *Kpna2^–/–^* mouse-derived zygotes. As shown in Figure 6, A and B, either human or mouse *KPNA2* cRNA could successfully rescue the phenotype of embryo arrest at concentrations of 1,000 ng/μL. More than half of the rescued embryos were able to develop to the blastocyst stage, showing the feasibility for this rescue strategy. Next, we tested to determine whether supplementation of *KPNA7* cRNA could also rescue the phenotype of embryo arrest in *Kpna2^–/–^* mouse-derived zygotes. To this end, cRNAs of human and mouse *KPNA7* were microinjected. At concentrations of 1,000 ng/μL, no arrested embryos were rescued in either group. When the concentration was increased to 3,000 ng/μL, more than half of the h*KPNA7* cRNA–injected embryos developed to the blastocyst stage and the transport of RSL1D1 into the nucleus increased significantly (Figure 6, C and D, and Supplemental Figure 8, A and B). However, in the m*KPNA7* cRNA–injected group, most embryos did not overcome the 2-cell stage (Figure 6, C and D). Unfortunately, preliminary attempts to transfer rescued blastocysts failed to establish pregnancy in foster female mice. The successful rescue using h*KPNA7* cRNA injection supports the conclusion that hKPNA7 and mKPNA2 play equivalent roles during embryonic development.

## Discussion

In this study, we used WES and population-based genetic burden analysis in our cohort of patients with the sporadic form of PREMBA and identified the causative gene *KPNA7*. When combined with the known gene *PADI6*, biallelic variants in these 2 genes could explain approximately 3.6% of our cohort (22 out of 606 affected individuals), underlining the significance of clinical screening for such recessive monogenic diseases. The existence of the *KPNA7* recurrent variant and the consistent phenotype in most patients strengthened the attribution of a genetic contribution of *KPNA7* to human PREMBA. A series of in vitro and in vivo studies further confirmed the crucial role of hKPNA7 and corresponding mKPNA2 during embryonic development.

Translocation of nuclear proteins from the cytoplasm to the nucleus is carried out by karyopherins, of which the karyopherin α/β system is the best-characterized import pathway for cNLS-cargo (15, 23). KPNA7 is the most abundant karyopherin α subtype in human oocytes and early embryos, and we found that the reduced protein levels of mutant KPNA7 and the impaired transport activity interrupted the nuclear import of certain substrates that are essential for the initiation of ZGA. To date, several genes and pathways involved in embryo arrest have been reported in mice, such as SCMC proteins (24), molecules participating in oocyte-to-embryo transition (25, 26), and ZGA (27). However, in humans, the limited genetic factors identified were mainly focused on SCMC proteins. Our present study uncovers a mechanism in addition to common SCMC protein defects, suggesting that deficiency in karyopherin α or molecules related to this pathway may also result in human PREMBA.

Differentiated function of the same gene in human and mouse oocytes has been observed in our previous studies. For example, mutant *PANX1* in human oocytes causes oocyte death, while oocytes of *Panx1* mutant mice are normal (28), and *TUBB8* is a primate-specific gene that is important for human oocyte spindle assembly (29), while the most abundant β-tubulin in mouse oocytes is *Tubb4b*. In this study, we showed that hKPNA7 deficiency and mKPNA7 depletion resulted in distinct phenotypes in humans and mice, respectively. *KPNA7* is the highest expressed karyopherin α family member in human oocytes and showed strong interactions with hRSL1D1. However, the expression of mouse *Kpna2* and its corresponding protein-binding capacity to mRSL1D1 was much higher than that of mouse *Kpna7*. Therefore, impairment of hKPNA7 results in human PREMBA, while mKPNA7 deletion has no obvious effect on female reproduction in our mouse model. In contrast, *Kpna2^–/–^* mice show a phenotype of early embryonic development, which can be rescued by microinjection of hKPNA7 cRNA. Thus, the function of mKPNA2 is equivalent to the role of hKPNA7 during early embryonic development, and this demonstrates an evolutionary difference between species in the roles of karyopherin α family members in early embryonic development. Notably, hKPNA2 and mKPNA2 also share highly conserved function because hKPNA2 can also rescue the phenotype of embryo arrest in *Kpna2^–/–^* mice. However, we have not demonstrated the pathogenic role of mutant *KPNA2* in our infertile cohorts. This may be due to the relatively low expression of *hKPNA2* or to functional redundancy compared with h*KPNA7* in human oocytes.

Although our C57BL/6 and ICR *Kpna7^–/–^* mice showed normal fertility, in another study, *Kpna7^–/–^* females were found to be subfertile (30). Different gene disruption strategies or different feeding environments might account for this divergence (31, 32). Unexpected transcripts or different gut microbiomes might also result in diverse phenotypes. However, the observation of normal fertility in our study and a weak reproductive phenotype in the previous study (30) suggests less importance of KPNA7 in mouse embryonic development compared with that of humans. In addition, regarding the *Kpna2-*KO models, C57BL/6 mice showed more severe defects, including reproductive deficiency, compared with mice on an ICR background. It was difficult to acquire C57BL/6 *Kpna2^–/–^* females, and postnatal females were weak and had small ovaries, while ICR *Kpna2^–/–^* females were normal and ovulated oocytes from ICR *Kpna2^–/–^* females could be retrieved. This is consistent with the phenomenon that the phenotypes of mouse models can vary among different backgrounds (16). Modifiers or protectors in one background may attenuate disease severity, while cooperators in other strains may increase disease severity. In our RNA-Seq analysis of *Kpna2^–/–^* mice, we used in vitro–fertilized 2-cell embryos. It has been reported that oocytes from mutant mice might be more sensitive to ovarian stimulation during IVF than those from WT females (33). So there exists a possibility that in vitro manipulation might lead to some adverse effects on gene expression in 2-cell embryos from *Kpna2^–/–^* females. However, the same culture condition and operations would partially reflect the real differentially expressed genes.

In addition, variants in genes related to nuclear import receptors have been reported to cause a variety of diseases, including limb-girdle muscular dystrophy (34), syndromic thoracic aortic aneurysm (35), and developmental delays and neurologic deficits (36). These receptors were restricted to karyopherin β. A previous study reported that compound variants in *KPNA7* were associated with infantile spasms and cerebellar malformation in a single family with 2 patients, but considering the very low expression level of *KPNA7* in the brain, the causal relationship between *KPNA7* variants and clinical presentation could not be definitively established (37, 38). Here, we confirmed the role of KPNA7 in human early embryonic development, and this finding expands human karyopherin-related disorders to include PREMBA and female infertility and establishes human disease caused by abnormality in karyopherin α.

In conclusion, we discovered pathogenic variants in the gene *KPNA7* responsible for human PREMBA. Our findings uncovered a mechanism in which karyopherin α, which regulates nucleocytoplasmic transport, plays crucial roles in human preimplantation embryonic development. We believe this is the first use of population-based screening for genetic factors contributing to human PREMBA. This finding will provide genetic diagnostic markers for the disease and potential therapeutic targets for the future treatment of infertile females with *KPNA7* pathogenic variants.

## Methods

### Study design.

The overall objective of this study was to identify underlying genetic causes and pathological mechanisms of PREMBA. Using WES data from 606 independent PREMBA patients and 2,813 controls, we performed a genetic burden analysis and identified 1 candidate recessive gene, *KPNA7*, in 10 independent families with 4 missense variants and 1 nonsense variant. The effect of *KPNA7* variants was assessed by Western blotting and immunoprecipitation in HEK293T cells, pull-down with purified proteins, and nuclear transport assays in semi-intact HeLa cells. The phenotype of human PREMBA was recapitulated by KO of the corresponding mouse model. The phenotype of the *Kpna2^–/–^* mouse model was rescued by injection of h*KPNA2*, h*KPNA7*, and m*Kpna2* cRNA. *Kpna2^–/–^* zygotes were randomized to be injected with cRNAs. Blinding was used for data analysis and in vitro microinjection. Experimental replicates and numbers of mice used for fertility evaluation varied in different experiments and are specified in the figure legends.

### Human subjects.

Independent patients with PREMBA and healthy controls were recruited from Shanghai Ninth Hospital, affiliated with Shanghai Jiao Tong University; Northwest Women’s and Children’s Hospital; Jiangsu Province Hospital; Shanghai Ji’ai Genetics and IVF Institute; Henan Provincial People’s Hospital; the Reproductive and Genetic Hospital of CITIC-Xiangya; and Nanchang Reproductive Hospital.

### Human embryo culture and phenotype evaluation.

Transvaginal oocyte retrieval was performed, and the cumulus-oocyte complex was isolated from the patients’ follicular fluid. In an ICSI cycle, hyaluronidase digestion treatment was performed to remove the cumulus cells surrounding the oocyte before injection. After IVF/ICSI, the oocytes were cultured in a humid atmosphere of 5% O_2_ and 5% CO_2_ at 37°C. Fertilization occurred within 14 to 16 hours after ICSI treatment. Normally fertilized oocytes continued to be cultured, and embryo quality was evaluated 3 days after fertilization. The developmental stage of the embryos was observed by light microscopy and time-lapse microscopy. Embryo evaluation was performed following the criteria of the Istanbul Consensus Workshop on Embryo Assessment (39).

### WES and variant calling.

Genomic DNA was extracted from peripheral blood leukocytes using the ETP-300 Nucleic Acid Extractor (Enriching). Exons and splice sites were captured using the SeqCap EZ Human Exome Kit (Roche), and sequencing was performed on an Illumina HiSeq 3000 platform. The Illumina lane-level fastq files were aligned to the hg19 reference genome with Burrows-Wheeler alignment (BWA) (40). GATK was used to recalibrate base quality scores, realign indels, remove duplicates, and call variants (41). Single nucleotide variants and indels were annotated by ANNOVAR software (42), including the dbSNP (43), gnomAD (44), and ExAC (45) databases.

### Variant filtering and assessment.

We filtered for rare variants that were well covered in both patient and control cohort sequencing with the following criteria: (a) minor allele frequency (MAF) less than 1% in the ExAC and gnomAD databases; (b) GATK variant quality score recalibration of “pass”; (c) minimum sequencing depth of 10 reads; (d) genotype quality score greater than 20 and alternate allele ratio greater than 40%; (e) predicted to be nonsynonymous or protein-truncating variants (referring to stopgain, splicing, and frameshift variants); (f) predicted to be putatively damaging (SIFT, PolyPhen-2); and (g) high mRNA expression in both human and mouse oocytes and preimplantation embryonic development according to our in-house RNA-Seq data (fragments per kilobase million [FPKM] > 10).

### Gene-based burden test of recessive diplotypes.

Existing research results suggest that recessive variants are widely implicated in Mendelian diseases such as galactosemia (46) and osteopetrosis (47), and many of the genes underlying complex diseases show loss-of-function effects in recessive patterns. Therefore, there was a high probability that the phenotype of embryo arrest would be inherited as a recessive risk trait. Thus, we prioritized an exome-wide, gene-based scan of recessive models to evaluate the enrichment of putative functional variants in PREMBA patients compared with control subjects. The total numbers of the 606 patients and 2,813 controls with qualifying variants in each gene using a recessive inheritance model were calculated, and a Fisher’s exact test was performed in R. The results were visualized as a Manhattan and quantile-quantile (QQ) plot using the R package qqman. The variation data reported in this paper have been deposited in the Genome Variation Map in the National Genomics Data Center, China National Center for Bioinformation/Beijing Institute of Genomics, Chinese Academy of Sciences (GVM000416 and GVM000417).

### Gene expression analysis.

Total RNA from human and mouse germinal vesicle (GV), MI, and MII stage oocytes, embryos, blastocysts, granulosa cells, mature sperm, and 3- to 4-month fetal tissues (including heart, liver, spleen, lung, kidney, brain, and spinal cord) was extracted with an RNeasy Mini Kit (QIAGEN) and was reverse transcribed with a PrimeScript RT Reagent Kit (Takara). The expression levels of *KPNA7*, *KPNA2*, and other related genes were examined using specific primers (Supplemental Table 6) and were normalized to the expression levels of *GAPDH* or *ACTB*. Real-time quantitative PCRs were performed in triplicate using a 7900HT Fast Real-Time PCR System (Applied Biosystems).

### Plasmid preparation.

Full-length human *KPNA7* transcript (GenBank NM_001145715.3), human *KPNA2* transcript (NM_002266.4), mouse *Kpna7* transcript (NM_001347531.1), mouse *Kpna2* transcript (NM_010655.3), and mouse *Rsl1d1* transcript (NM_025546.2) were amplified from human or mouse MII oocyte cDNA. The full-length *RSL1D1* human coding sequence was synthesized by GENEWIZ Biotechnology Co. Human *KPNA7* cDNA was cloned into the PCMV6 entry vector with a FLAG tag at the N-terminus or a GFP tag at the C-terminus. Mouse *Kpna7* cDNA was cloned into the PCMV6 entry vector with an N-terminal FLAG tag. Human *KPNA2* and mouse *Kpna2* were cloned into the pCMV6 entry vector with a C-terminal HA tag or N-terminal FLAG tag. Human *RSL1D1* and mouse *Rsl1d1* were cloned into pGEX4T-1, pCR3.1, and PCMV6 entry vectors with GST, fluorescent mClover3 tag at the N-terminus, and HA tag at the C-terminus, respectively. The plasmids pGEX6P-1-h-KPNA7, pGEX2T–importin β1, pGEX6P1–Ran, pGEX6P1–NTF2, and pGEX2T–SV40TNLS–GFP were obtained as described previously (11). Site-directed mutagenesis was performed with the KOD-Plus Mutagenesis Kit according to the manufacturer’s instructions (SMK-101, TOYOBO), and clones were confirmed by Sanger sequencing. Primers required for amplification and point mutations are listed in Supplemental Table 6.

### Cell culture and transfection.

HeLa and HEK293T cells were purchased from Cell Bank of the Chinese Academy of Sciences and were cultured in DMEM supplemented with 10% fetal bovine serum and 1% penicillin-streptomycin and incubated at 37°C and 5% CO_2_. Cells were seeded into 6 cm dishes or 24-well plates with coverslips 1 day before transfection. Plasmids were transfected into cells with PolyJet In Vitro DNA Transfection Reagent (Signagene). The culture medium was replaced 12 to 18 hours after transfection.

### Immunofluorescence.

HeLa cells transfected with KPNA7-GFP were fixed with 4% paraformaldehyde in PBS and incubated in a blocking solution (PBS, 1% BSA, and 0.1% Tween-20) for 1 hour. DNA was labeled with Hoechst 33342 solution (1:700 dilution, 561908, BD). Confocal images of KPNA7 localization were obtained with a Leica TCS SP8 microscope.

Zygotes and 2-cell embryos were fixed with 2% paraformaldehyde in PBS and permeabilized in PBS containing 0.5% Triton X-100, followed by incubation in a blocking solution (PBS, 1% BSA, and 0.1% Tween-20) for 1 hour. Zygotes were then incubated with anti-RSL1D1 (orb704476, Biorbyt) or anti-KPNA2 (ab70160, Abcam) antibodies at 1:400 dilution in 5% BSA at 4°C overnight and were visualized using laser scanning confocal microscopy (LSM880, Zeiss) after incubation with donkey anti-rabbit secondary antibody (A21206, Thermo Fisher) for 2 hours at room temperature.

### Western blot.

HEK293T cells were collected at 36 hours after transfection, and cells were lysed in radio-immunoprecipitation assay lysis buffer containing protease inhibitor cocktail (B14001, Bimake). Equal amounts of samples were electrophoresed on SDS-PAGE gels and transferred to nitrocellulose membranes. Membranes were blocked with 5% nonfat milk in PBS with 0.1% Tween 20 and then incubated with primary antibodies diluted in 5% BSA at 4°C overnight. The membranes were visualized using ECL Western Blotting Substrate (180-501, Tanon) after incubation with HRP-conjugated secondary antibody for 2 hours at room temperature. Digital images were obtained from a Tanon 5200s Imaging Workstation. The following primary antibodies were used for Western blots: rabbit antibody against KPNA7 (HPA031395, Sigma-Aldrich) at 1:1000 dilution, rabbit anti-FLAG (F7425, Sigma-Aldrich) at 1:3000 dilution, rabbit anti-HA (3724, Cell Signaling) at 1:2000 dilution, rabbit anti-vinculin (13901, Cell Signaling) at 1:3000 dilution, mouse anti-GST (A00865, GenScript) at 1:1000 solution, and rabbit anti-KPNA2 (ab70160, Abcam) at 1:1000 dilution.

### Immunoprecipitation.

HEK293T cells were cotransfected with KPNA2-HA (or RSL1D1-HA) and FLAG-KPNA7 plasmids. WT and mutant KPNA7 protein levels were adjusted to be equal. At 36 hours after transfection, cell proteins were extracted in NP-40 lysis buffer (50 mM Tris, 150 mM NaCl, 0.5% NP-40, pH 7.5) with 1% protease inhibitor cocktail (Bimake). pH was adjusted with the equipment METTLER TOLEDO, S210. FLAG-IgG Sepharose beads (Bimake) were added to total proteins and incubated at 4°C for 3 hours on a rotating wheel. The beads were washed using NP-40 buffer and then boiled with SDS loading buffer for Western blotting.

### Protein purification.

A single colony of BL21 (DE3) plysS transformed with each respective plasmid was cultured in Luria broth (LB) medium until OD_600_ 0.6 to 0.8 at 37°C. Then 0.3 mM isopropyl-1-thio-β-d-galactopyranoside (IPTG) was added to the culture to induce expression, followed by culture for 16 hours at 16°C. After centrifugation at 1,000*g*, the bacteria pellet was sonicated in lysis buffer (50 mM Tris-HCl, 500 mM NaCl, 1 mM EDTA [2 Na], 2 mM DTT, and protease inhibitor, pH 8.3). The supernatant after centrifugation was incubated with glutathione Sepharose (GE 17075601) in lysis buffer for 3 hours at 4°C. After washing the Sepharose beads, the proteins were eluted with elution buffer (100 mM Tris-HCl, 100 mM NaCl, 1 mM EDTA [2Na], 2 mM DTT, protease inhibitors, and 20 mM glutathione, pH 8.3). Eluted proteins were dialyzed against cleavage buffer (50 mM Tris-HCl, 150 mM NaCl, 1 mM EDTA, 1 mM DTT, pH 7.0) using a 3.5 to 5 kDa dialysis membrane overnight at 4°C. PreScission protease (GenScript) was used to cut the GST tag overnight at 4°C. Finally, purified proteins were dialyzed against transport buffer (20 mM HEPES, 100 mM CH3COOK, 2 mM DTT, and protease inhibitors, pH 7.3).

### Binding assays.

GST-SV40TNLS-GFP and GST-RSL1D1-GFP proteins were incubated with GST beads at 4°C for 1 hour in transport buffer containing 0.1% Triton X-100. After washing, the beads were incubated with cleaved WT and mutant KPNA7 at 4°C for 2 hours. The beads were then washed with transport buffer with 0.1% Triton X-100 and boiled with SDS loading buffer for Western blotting.

### In vitro nuclear transport assay.

To create RanGDP, cleaved Ran was incubated with 25 mM EDTA and 2 mM GDP (Sigma-Aldrich) on ice for 1 hour, followed by the addition of 50 mM MgCl_2_. HeLa cells were permeabilized with 40 μg/ml digitonin (Nacalai Tesque) on ice for 5 minutes. Then 10 μL reaction mix including 4 pmol GST-SV40TNLS-GFP, 6 pmol WT or mutant KPNA7, 4 pmol importin β1, 40 pmol RanGDP, 0.5 mM ATP (Wako), 20 U/ml creatine phosphokinase (Sigma-Aldrich), and 5 mM creatine phosphate (Sigma-Aldrich) was added to the permeabilized cells and incubated at 30°C for 30 minutes. The cells were then fixed with 3.7% formaldehyde and the fluorescence images were observed using a confocal microscope (Leica TCS SP8 II; Leica Microsystems). All images were collected under the same conditions as WT. The fluorescence intensities in the nucleus were identified by a region of interest in the Leica Application Suite X software.

### Generation of model mice.

A CRISPR/Cas9 system was used to generate knockin or KO mice. Cas9 and sgRNA were injected into the fertilized eggs of C57BL/6 mice. Positive F0 mice were identified by PCR and sequencing analysis. The stable inheritable positive F1 mouse model was obtained by mating F0 mice with C57BL/6J mice. Homozygous targeted mice were obtained by intercrossing heterozygous targeted mice. For *Kpna7* knockin mice, donor oligo with a CTG-to–TTC mutation for L222F and 120 bp homologous sequences on both sides of mutant site was coinjected with Cas9 and sgRNA. For *Rsl1d1*^OO–/–^ mice, targeting vector with LoxP site was coinjected with Cas9 and sgRNA. Homozygous *Rsl1d1* targeted mice were bred with Zp3-cre tool mice to generate *Rsl1d1*^OO–/–^ mice.

### IVF, microinjection, and mouse embryo culture.

Sperm were collected from the epididymis of 8- to 9-month-old male ICR mice and capacitated in EmbryoMax Human Tubal Fluid (HTF, Merck Millipore) for 40 minutes at 37°C and 5% CO_2_. The oocyte-cumulus complexes were collected from superovulated 5- to 6-week-old or 7- to 8-week-old female mice and placed in HTF at 37°C in an atmosphere of 5% CO_2_. An appropriate amount of capacitated sperm was added into the HTF liquid drops, which contained oocyte-cumulus complexes. After 6 hours, mouse zygotes were microinjected with siRNA or cRNA. siRNAs and cRNAs were as follows: *Rsl1d1* siRNA (si*Rsl1d1*, 5′–CCUCAGAUGUAUGUCUCUUTT–3′, 40 μM); synonymous mutated *Rsl1d1* cRNA (1,000 ng/μL); cRNA of mClover3-RSL1D1 (1,000 ng/μL) and H_2_B-mcherry (1,000 ng/μL); human *KPNA7* cRNA (3,000 ng/μL); mouse *Kpna7* cRNA (3,000 ng/μL); human *KPNA2* cRNA (1,000 ng/μL); and mouse *Kpna2* cRNA (1,000 ng/μL). After injection, zygotes were transferred to KSOM medium (Nanjing Aibei Biotechnology) for culture. Embryos were assessed at 24 hours, 48 hours, 60 hours, 72 hours, and 108 hours after fertilization. Fluorescence was observed 3 hours after injection for the RSL1D1 nuclear transport assays.

### PLA.

Duolink in Situ Orange Starter Kit for Goat/Rabbit Antibody (DUO92106, Sigma-Aldrich) was used for PLA. Assays were performed according to the manufacturer’s instructions. The PLA kit required different species as sources of primary antibodies, and our RSL1D1 and KPNA2 antibodies were both generated in rabbit. Thus, we tested the interaction of KPNA2 with overexpressed RSL1D1 instead of endogenous RSL1D1. Oocytes from WT and *Kpna2^–/–^* females were fertilized with sperm from WT males. Five hours after fertilization, cRNAs of mRSL1D1-HA were microinjected into zygotes. Ten hours after microinjection, zygotes were fixed in 2% paraformaldehyde in PBS, permeabilized in PBS containing 0.5% Triton X-100, blocked in Duolink blocking solution, and incubated with anti-HA (ab215069, Abcam) and anti-KPNA2 (ab70160, Abcam) antibodies at 1:400 dilution. A combination of secondary anti-rabbit/goat antibodies with PLA PLUS/MINUS probes was used, followed by hybridization, ligation, and amplification steps. PLA signals were visualized with laser scanning confocal microscopy (LSM880, Zeiss).

### Affymetrix cDNA microarray.

For 2-cell embryos, 24 hours after fertilization with sperm from WT males, 5 embryos from *Kpna2^+/–^* and *Kpna2^–/–^* female littermates were collected and lysed in triplicate. The NEBNext Single Cell/Low Input RNA Library Prep Kit (NEB, E6420) was used for ultra-low RNA reverse transcription, amplification, and Illumina library preparation. Sequencing was performed on an Illumina HiSeq 3000 platform. Sequencing reads were filtered, trimmed, and then mapped to the Ensembl gene annotation and the mouse genome assembly GRCm38 using STAR aligner. Differentially expressed gene (DEG) analysis was performed with the R package DESeq2 (adjusted *P* < 0.05, fold-change ≥ 1.5, and FDR < 0.05). The R package clusterProfiler was used to perform Gene Ontology analyses with adjusted *P* < 0.05. Large data sets for microarrays were deposited as noted previously in the public repository Genome Variation Map in the National Genomics Data Center, China National Center for Bioinformation/Beijing Institute of Genomics, Chinese Academy of Sciences.

### BrUTP incorporation.

At 5 hours and 24 hours after fertilization, WT and *Kpna2*-KO zygotes and 2-cell embryos were incubated in 5-ethynyl uridine (E10345, ThermoFisher). At 5 hours for zygotes and 1 hour for 2-cell embryos after incubation, embryos were fixed with 2% paraformaldehyde for 30 minutes and permeabilized in PBS containing 0.5% Triton X-100 and labeled with Alexa Fluor 594 Azide (Thermo Fisher, A10270) using Click-iT Cell Reaction Buffer Kit (Thermo Fisher, C10269).

### Data and materials availability.

The data sets generated and/or analyzed during the current study are available from the corresponding author on reasonable request.

### Statistics.

Statistical analysis was performed using GraphPad Prism. Values were analyzed by 2-tailed Student’s *t* test for 2 experimental groups or by 1-way ANOVA for more than 2 groups. A *P* value of less than 0.05 was considered significant.

### Study approval.

This study was approved by the collaborating hospitals and the Ethics Committee of the Medical College of Fudan University. Written, informed consent was obtained from all participants. Mice were maintained and euthanized according to procedures approved by the Experimental Animal Ethics Committee of Fudan Medical College.

## Author contributions

Wenjing Wang, QS, and L Wang contributed to conception and design. Wenjing Wang and YM contributed to experimental work and interpretation of data. BC and Qun Li performed data and statistical analysis. JS, FD, WZ, LY, LL, YX, L Wu, XM, JF, BL, ZY, RS, XX, JM, Z Zhang, LZ, Weijie Wang, Z Zhou, JD, TW, Qiaoli Li, LJ, LH, XS, GL, and YK collected the samples. JS, FD, and YK organized the medical records. QS and L Wang were responsible for overall supervision. Wenjing Wang, YX, and BC drafted the manuscript, which was revised by QS, L Wang, and YM. All authors read and approved the final manuscript. The authorship order among co–first authors was assigned according to the amount of work.

## Supplementary Material

Supplemental data

## Version 1 01/17/2023

Electronic publication
