# Supplementary material for: Karyopherin α deficiency contributes to human preimplantation embryo arrest
Source: J Clin Invest. 2023 Jan 17;133(2):e159951. doi: 10.1172/JCI159951 (PMC9843055; doi:10.1172/JCI159951)
Supplement: Supplemental data [file jci-133-159951-s007.pdf]

## Supplementary Figures

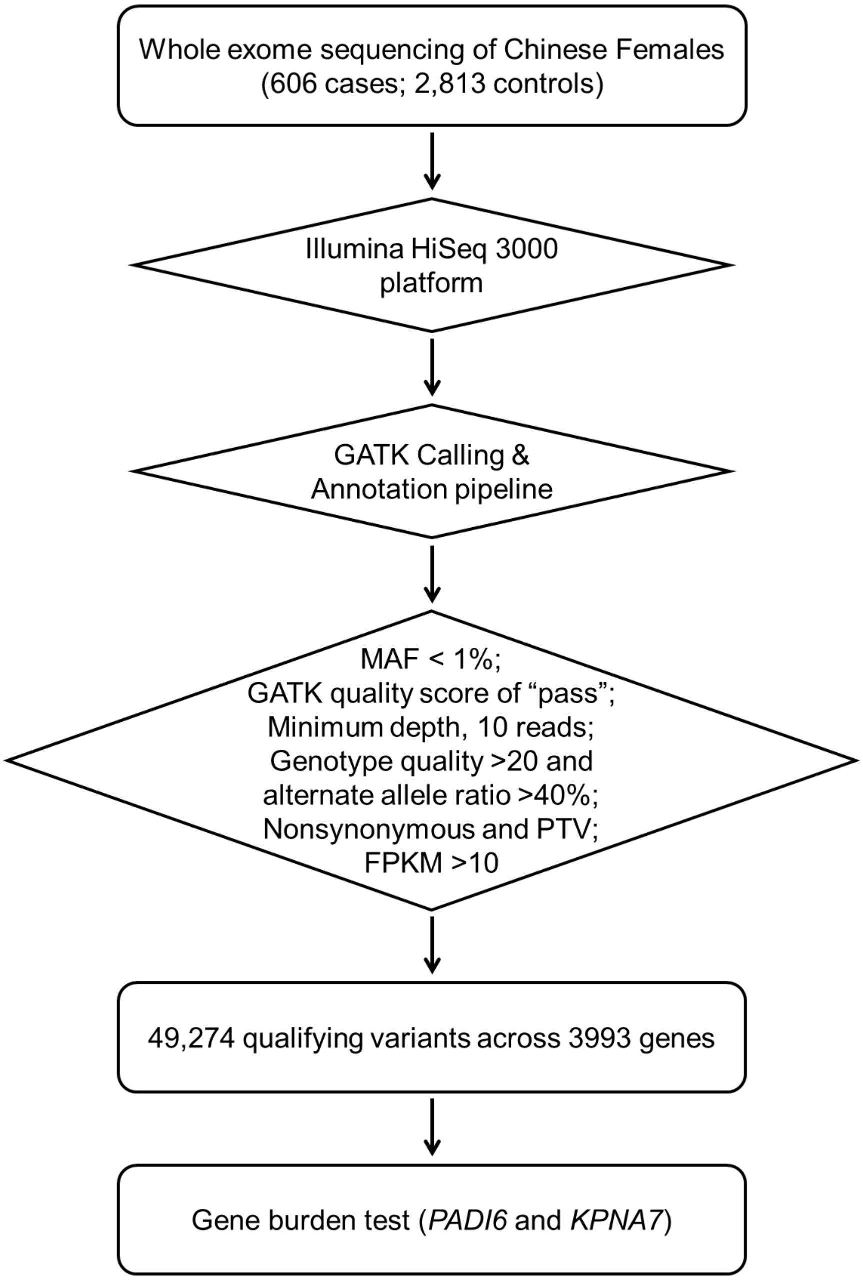


**Supplemental Figure 1. Burden testing scheme**

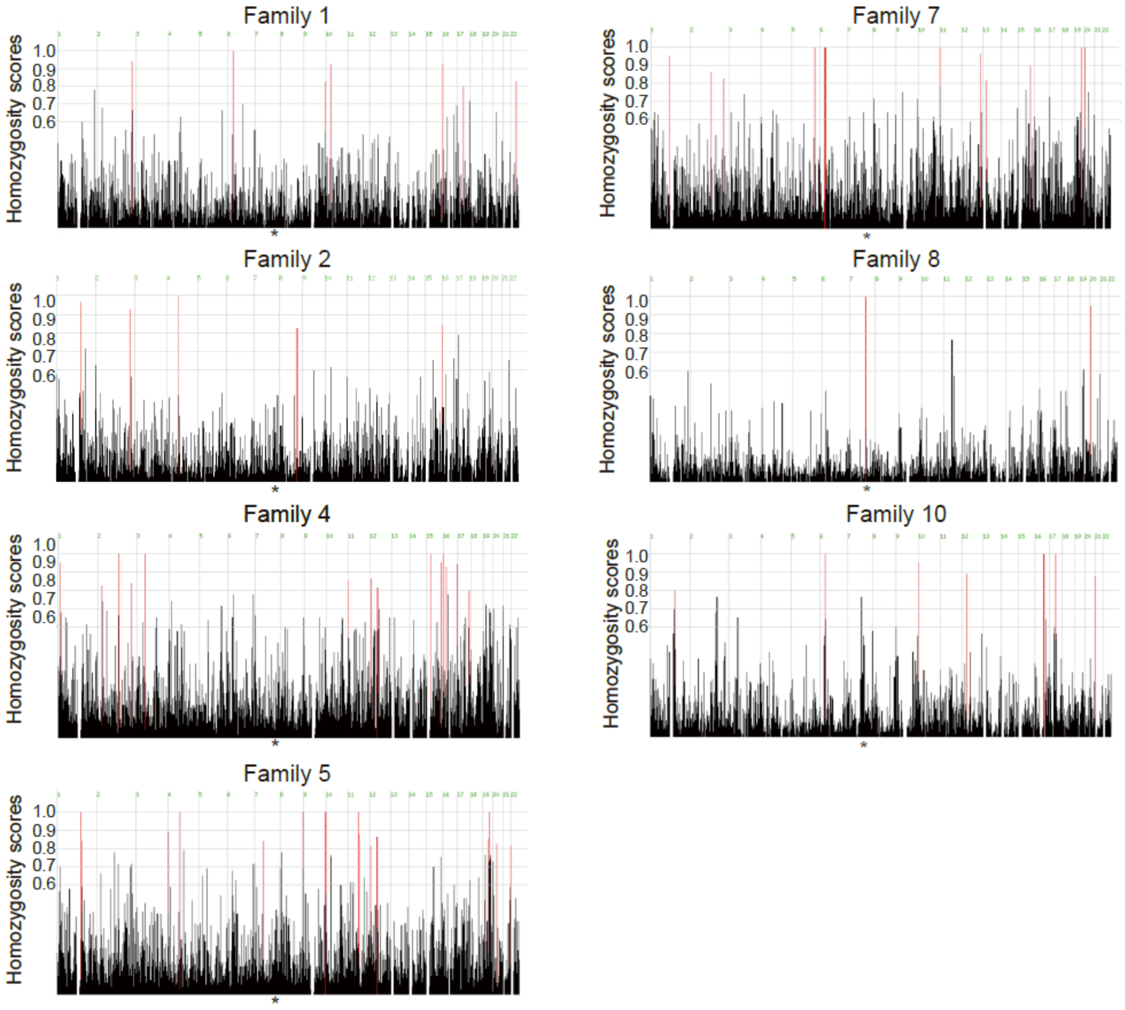


**Supplemental Figure 2. Homozygosity mapping of patients carrying homozygous *KPNA7* variants**

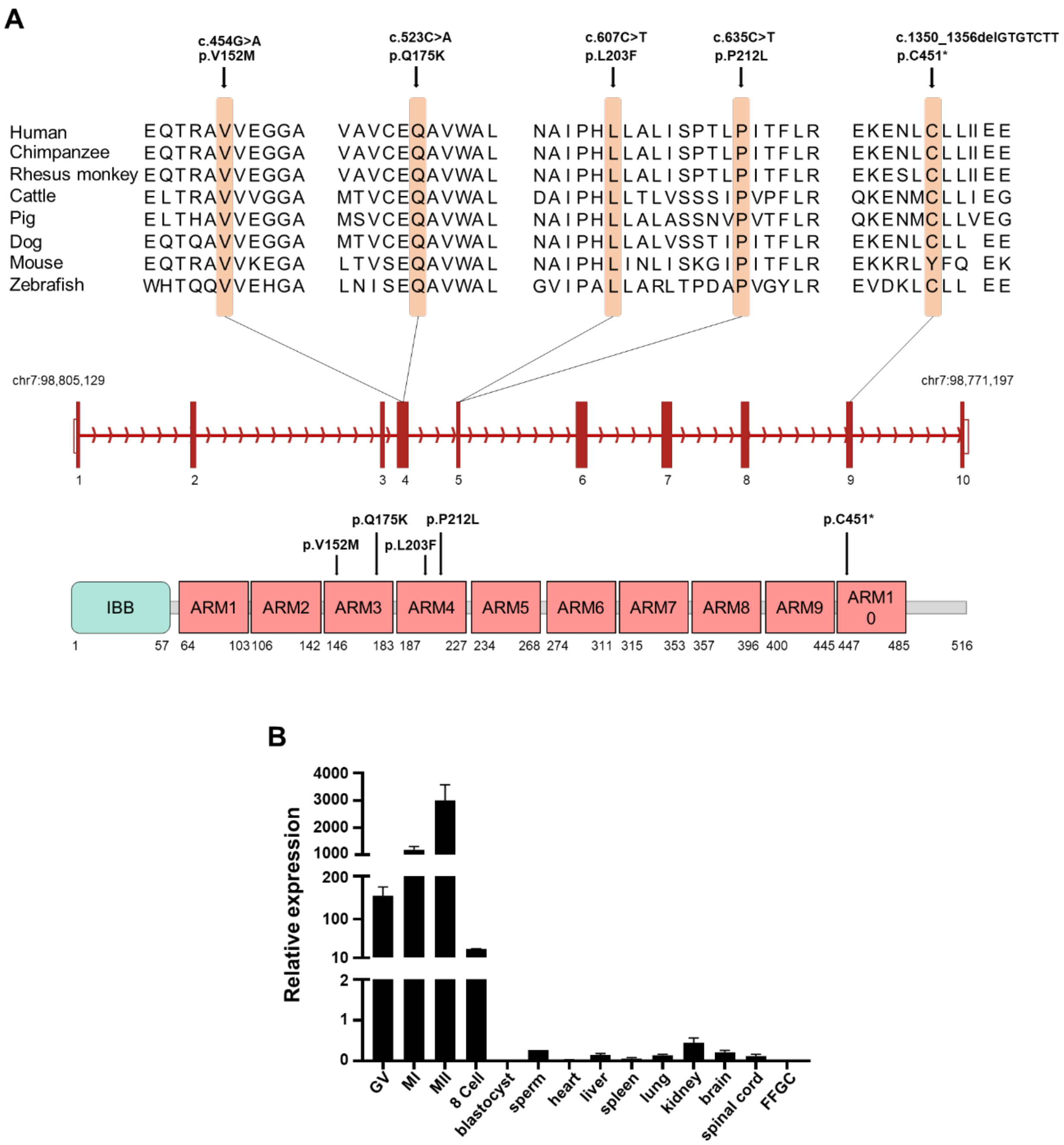


**Supplemental Figure 3. Structure, conservation, and expression pattern of *KPNA7***

(**A**) The structure and protein domain architecture of KPNA7 and the location and conservation of the residues that were changed by the variants in *KPNA7*. IBB, importin

β binding; ARM, armadillo. (**B**) The relative expression of *KPNA7* by Real-time qRT-PCT in developing human oocytes, early embryos, and several somatic tissues.

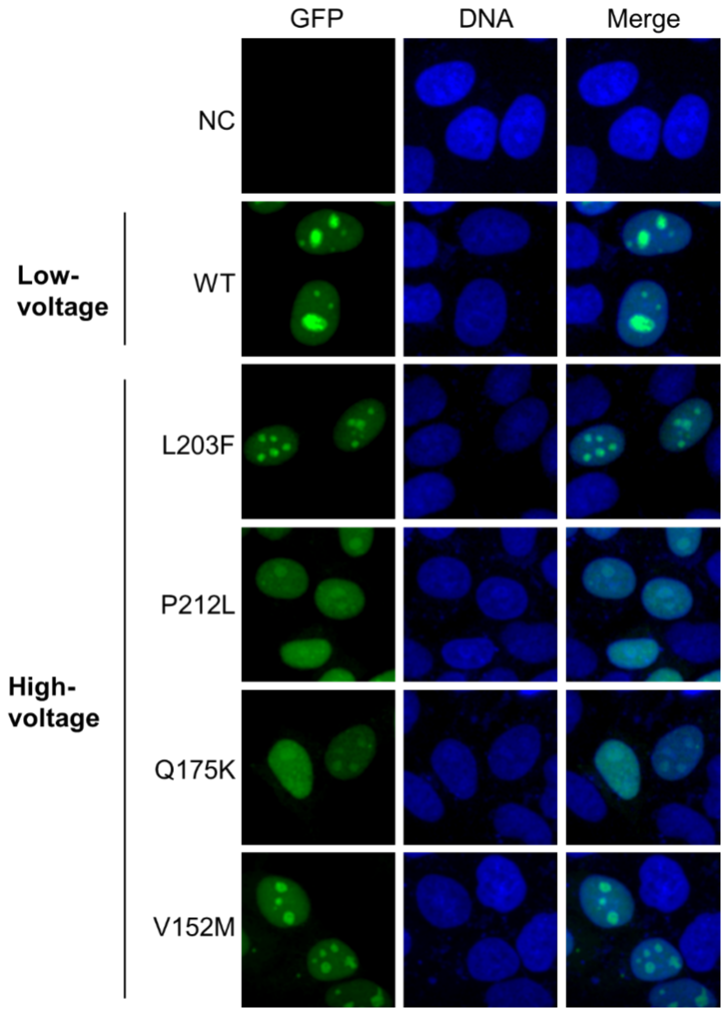


**Supplemental Figure 4. Localization of GFP-tagged wild-type and mutant KPNA7 constructs in HeLa cells**

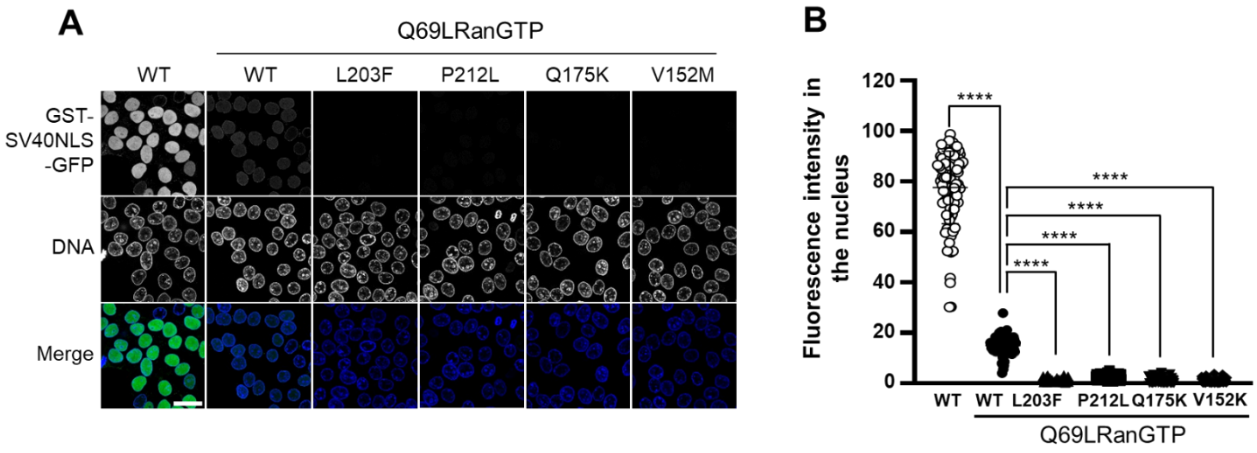


**C**

**Ratio of average fluorescence intensity in the nucleus**

| | RanGDP | Q69LRanGTP |
|---|---|---|
| WT/L203F | 1.561001 | 14.47495 |
| WT/P212L | 2.273832 | 5.140267 |
| WT/Q175R | 3.82446 | 18.97096 |
| WT/V152M | 4.228425 | 22.66214 |

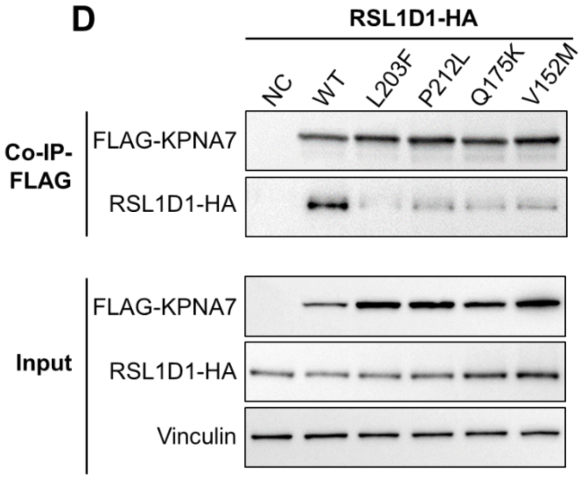


**Supplemental Figure 5. Effect of variants on the binding ability and transport capacity of KPNA7**

(**A**) In vitro nuclear transport assays of purified wild-type and missense mutant KPNA7 with the transport substrate GST-SV40TNLS-GFP when added Q69RanGTP. GTP fixed Ran affected transport ability of KPNA7. DNA was stained with DAPI. Scale bar = 30 μm. (**B**) Quantitative analysis of KPNA7 transport capacity in (**A**). A total of 100 cells were counted. Data are shown as individual values with means ± s.d. One-way ANOVA. (**C**) Ratio of average fluorescence intensity in the nucleus by comparing wild-

type group to mutant group. Functions of mutant KPNA7 were more sensitive to Q69LRanGTP. (**D**) Co-immunoprecipitation from HEK293T cells expressing FLAG-tagged wild-type or missense mutant KPNA7 and HA-tagged RSL1D1 with anti-FLAG beads. Missense alterations affect the interaction between KPNA7 and RSL1D1.

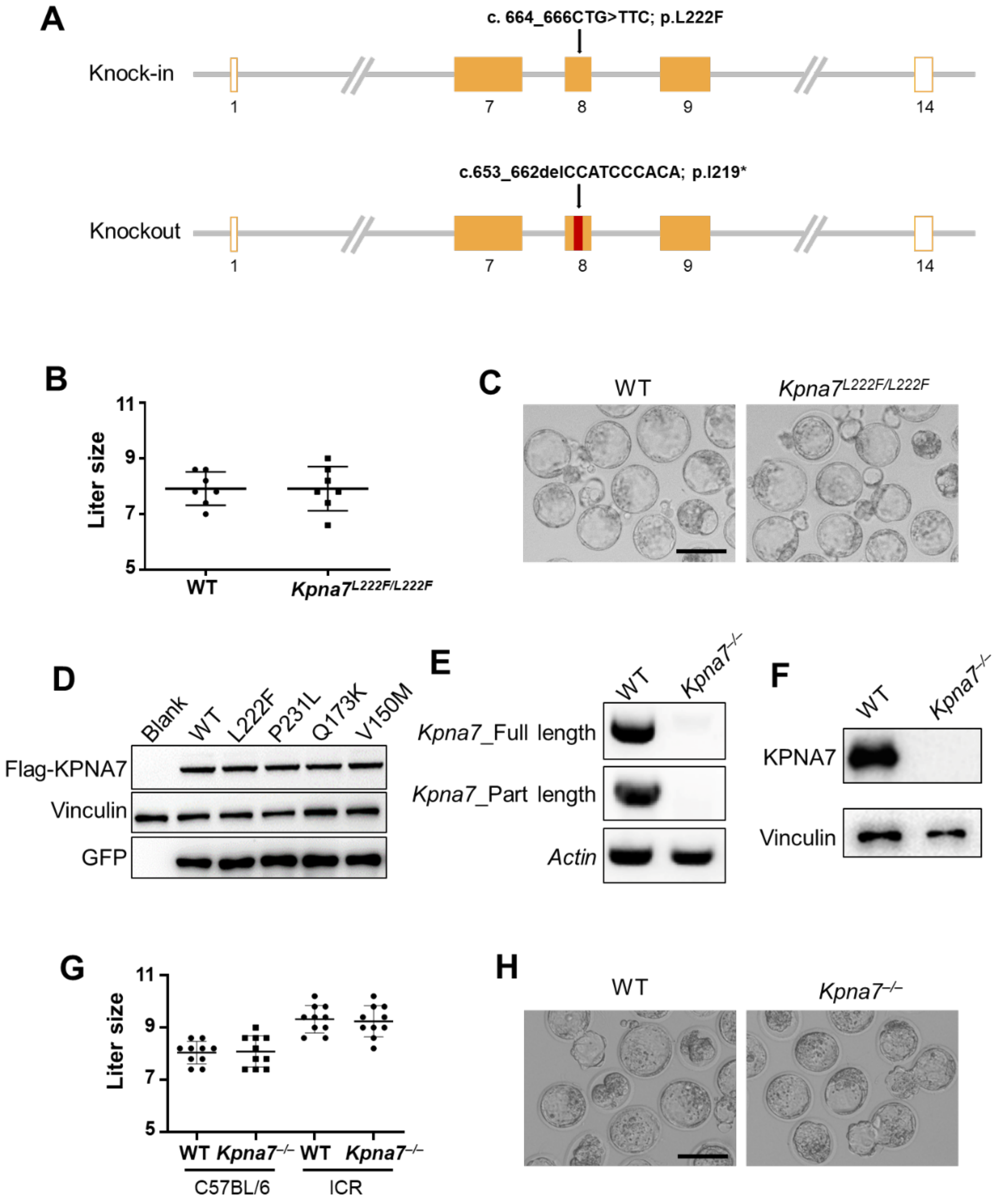


**Supplemental Figure 6. Fertility of *Kpna7* knock-in and knockout female mice.** (**A**) Schematic diagram of *Kpna7* L222F knock-in and knockout. The red box indicates the knockout area. (**B**) Statistical analysis of the reproductive ability of wild-type and *Kpna7*$^{\mathrm{L222F/L222F}}$ mice in the C57BL/6 background. $n$ = 7. Data are shown as individual values with means ± s.d. (**C**) In vitro embryonic development phenotype of wild-type

and *Kpna7*$^{L222F/L222F}$ mice. Scale bar = 100 μm. (**D**) Immunoblot of wild-type and missense mutant mouse KPNA7 proteins in HEK293T cells. Due to similar protein size of KPNA7 and GFP, blots were run in parallel. (**E**) RNA level of *Kpna7* in wild-type and *Kpna7*$^{-/-}$ oocytes. *Kpna7* was completely knocked out in *Kpna7*$^{-/-}$ oocytes. (**F**) Immunoblot of wild-type and *Kpna7*$^{-/-}$ oocytes. (**G**) Statistical analysis of the reproductive ability of *Kpna7*$^{-/-}$ mice in the C57BL/6 and ICR background. $n = 10$. Data are shown as individual values with means ± s.d. (**H**) In vitro embryonic development phenotype of wild-type and *Kpna7*$^{-/-}$ mice. Scale bar = 100 μm.

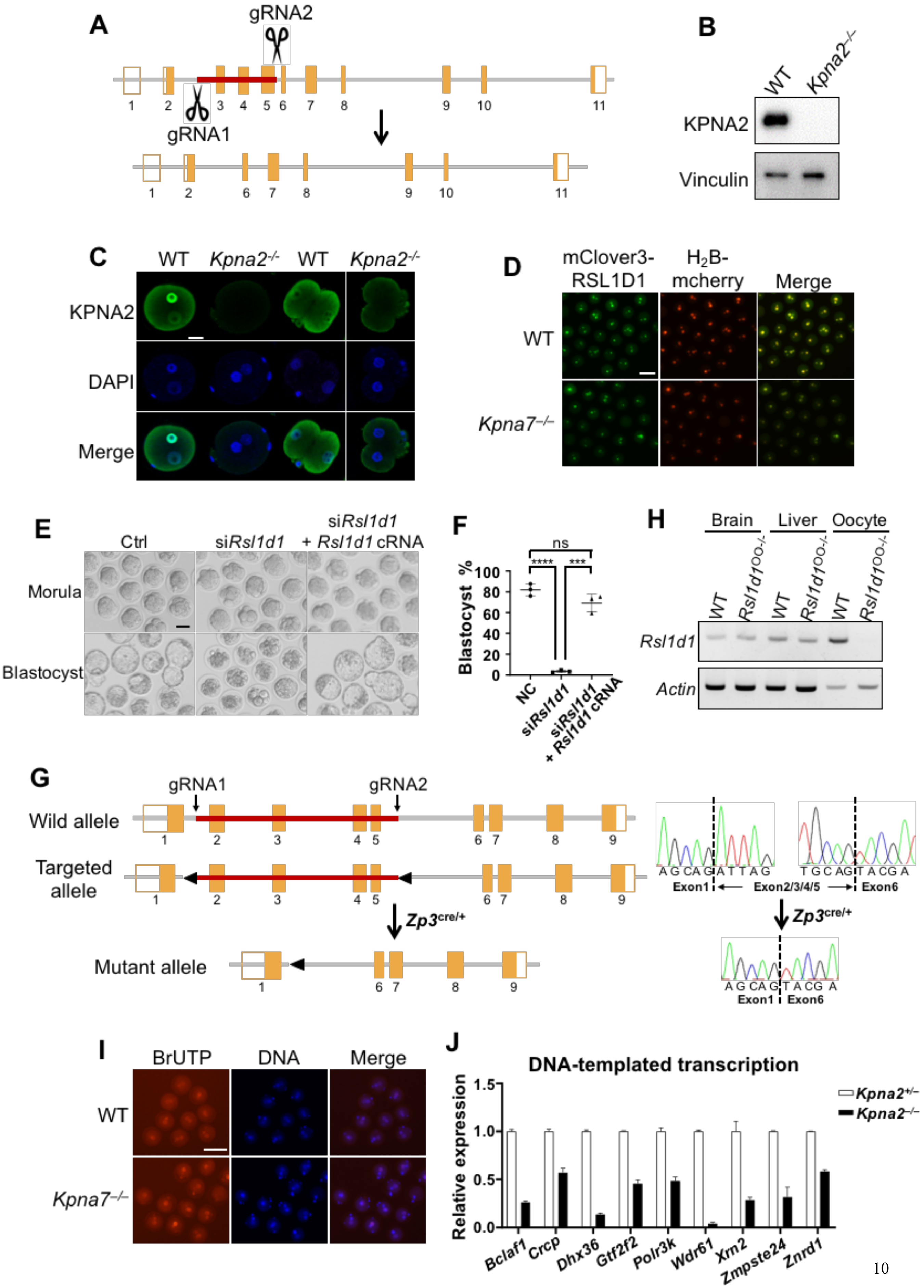
A
gRNA2
gRNA1
B
WT
Kpna2−/−
KPNA2
Vinculin
C
WT
Kpna2-/-
KPNA2
DAPI
Merge
D
mClover3-RSL1D1
H2B-mcherry
Merge
WT
Kpna7−/−
E
Ctrl
siRsl1d1
siRsl1d1 + Rsl1d1 cRNA
Morula
Blastocyst
F
ns
****
***
Blastocyst %
NC
siRsl1d1
siRsl1d1 + Rsl1d1 cRNA
H
Brain
Liver
Oocyte
WT
Rsl1d1OO-/-
Rsl1d1
Actin
G
Wild allele
Targeted allele
Mutant allele
Zp3cre/+
Exon1
Exon2/3/4/5
Exon6
I
BrUTP
DNA
Merge
WT
Kpna7−/−
J
DNA-templated transcription
Relative expression
Kpna2+/−
Kpna2−/−
Bclaf1
Crcp
Dhx36
Gtf2f2
Polr3k
Wdr61
Xrn2
Zmpste24
Znrd1

**Supplemental Figure 7. Infertility of *Kpna2*$^{-/-}$ females and fertility of *Kpna7*$^{-/-}$ females.**

(**A**) Schematic diagram of *Kpna2* knockout mice. The red box indicates the knockout area. (**B**) Immunoblot of wild-type and *Kpna2*$^{-/-}$ oocytes. (**C**) The expression and localization of endogenous KPNA2 in 1-cell and 2-cell embryos from wild-type and *Kpna2*$^{-/-}$ females fertilized with wild-type sperms in vitro. Scale bar = 20 μm. (**D**) Localization of RSL1D1 at 3 h after injection of mClover3-RSL1D1 cRNA into zygotes from wild-type and *Kpna7*$^{-/-}$ females. Scale bar = 100 μm. (**E**) In vitro embryonic development phenotype after *Rsl1d1* knockdown in mice and phenotypic rescue by mouse *Rsl1d1* cRNA injection into 2 PN zygotes. (**F**) Quantification of (**E**). Data are shown as individual values with means ± s.d. $n = 3$ biological replicates. Unpaired two-sided *t*-test. (**G**) Schematic diagram and Sanger sequencing confirmation of mice with an oocyte-specific knockout of *Rsl1d1*. The red box indicates the knockout area. (**H**) RNA level of *Rsl1d1* in oocytes and somatic tissues of wild-type and *Rsl1d1*$^{OO-/-}$ mice. *Rsl1d1* was completely knocked out in *Rsl1d1*$^{OO-/-}$ oocytes, while in somatic tissues, RNA level of *Rsl1d1* did not change. (**I**) Incorporation of BrUTP to mark the synthesis of nascent transcripts in zygotes from wild-type and *Kpna7*$^{-/-}$ females. Scale bar = 100 μm. (**J**) Real-time qRT-PCR analyses of down-regulated genes in 2-cell embryos from *Kpna2*$^{-/-}$ females compared to embryos from *Kpna2*$^{+/-}$ females. Data are shown as mean ± s.d.

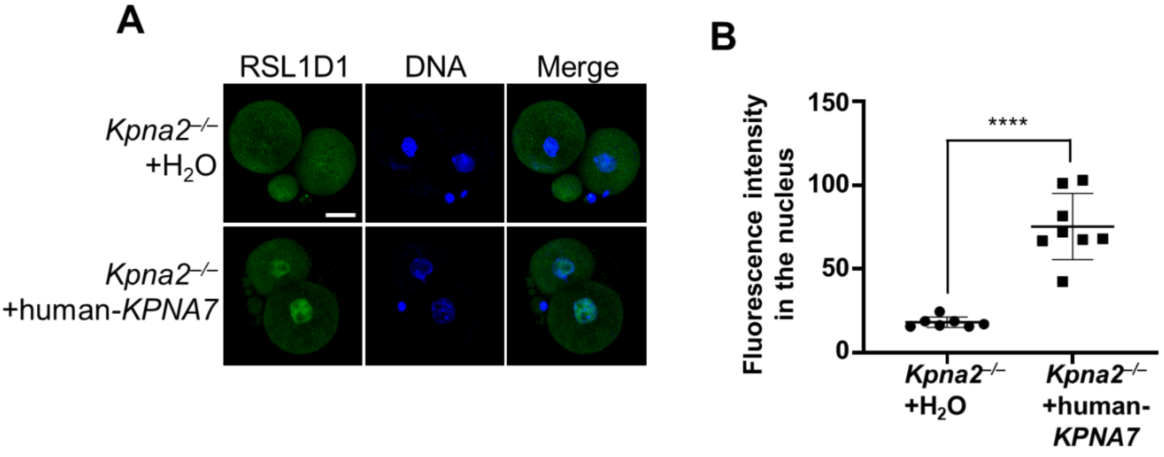


**Supplemental Figure 8. Phenotypic rescue by cRNA injection.**

(**A**) Molecular rescue by cRNA injection of human *KPNA7* into *Kpna2*$^{-/-}$ zygotes. After injection, the nuclear localization intensity of endogenous RSL1D1 in 2-cell embryos was stronger. (**B**) Fluorescence intensity of RSL1D1 in the nucleus of (**A**). Scale bar = 20 μm. Data are shown as individual values with means ± s.d. $n$ = 2 biological replicates. Unpaired two-sided $t$-test.

## Supplementary Tables

**Supplemental Table 1. Overview of the *KPNA7* variants**

| Genomic Position on Chr 7 (bp) | cDNA Change | Protein Change | Variant Type | Inheritance[c] | Phenotype | ExAC[b] | GnomAD[b] | SIFT[a] | PPH2[a] |
|---|---|---|---|---|---|---|---|---|---|
| 98,790,671 | c.C607T | p.L203F | missense | AR | embryo arrest | 1.41E-04 | 3.21E-04 | D | P |
| 98,790,643 | c.C635T | p.P212L | missense | AR | embryo arrest | 4.75E-05 | 6.02E-05 | D | P |
| 98,792,723 | c.C523A | p.Q175K | missense | AR | embryo arrest | NA | NA | D | P |
| 98,792,792 | c.G454A | p.V152M | missense | AR | embryo arrest | NA | 0.00003201 | D | P |
| 98,775,650 | c.1350_1356delGTGTCTT | p.C451* | frameshift | AR | embryo arrest | NA | NA | NA | NA |

[a]Variant assessment by SIFT and PolyPhen-2 (PPH2). D, damaging; P, probably damaging.

[b]Frequency of corresponding variants in ExAC Browser and gnomAD. NA, not available.

[c]AR, Autosomal recessive.

**Supplemental Table 2. Clinical characteristics of the patients with *KPNA7* variants**

| Family | Age (years) | Duration of infertility (years) | IVF & ICSI cycles | Total oocytes retrieved | MII oocytes with normal morphology | Normal fertilized oocytes | Normal cleaved embryos | Usable embryos at day 3 | Usable blastocysts | Outcome of embryo transfer |
|---|---|---|---|---|---|---|---|---|---|---|
| 1 | 39 | 12 | ICSI | 20 | 20 | 18 | 18 | 0 | 0 | / |
| | | | ICSI | 10 | 8 | 6 | 6 | 0 | 0 | / |
| | | | ICSI | 2 | 2 | 2 | 2 | 0 | 0 | / |
| | | | ICSI | 37 | 28 | 19 | 18 | 2 | 0 | Failure |
| | | | ICSI | 28 | 16 | 16 | 14 | 2 | 0 | / |
| 2 | 31 | 3 | IVF | 19 | 17 | 16 | 12 | 2 | 0 | / |
| | | | ICSI | 22 | 20 | 14 | 13 | 5 | 0 | / |
| 3 | 37 | 7 | IVF | 5 | 2 | 2 | 2 | 0 | / | / |
| | | | IVF | 8 | 4 | 4 | 3 | 0 | / | / |
| | | | IVF | 10 | 8 | 8 | 8 | 2 | 0 | Failure |
| | | | IVF | 11 | 11 | 11 | 11 | 0 | / | / |
| | | | IVF | 1 | 1 | 1 | 1 | 0 | / | / |
| | | | IVF | 17 | 16 | 16 | 15 | 1 | / | / |
| | | | ICSI | 10 | 8 | 8 | 6 | 0 | / | / |
| | | | ICSI | 10 | 9 | 8 | 7 | 2 | 0 | Failure |
| | | | ICSI | 10 | 6 | 6 | 5 | 0 | / | / |
| | | | ICSI | 15 | 10 | 10 | 9 | 2 | / | / |
| | | | ICSI | 22 | 15 | 15 | 11 | 4 | 0 | Failure |

| | | | | | | | | | | |
|---|---|---|---|---|---|---|---|---|---|---|
| 4 | 39 | 8 | ICSI | 8 | unknown | 0 | / | / | / | / |
| | | | ICSI | 12 | 6 | 4 | 4 | 0 | / | / |
| 5 | 34 | 8 | IVF | 10 | 8 | 8 | unknown | 4 | 0 | / |
| | | | IVF | 9 | 6 | 6 | unknown | 0 | / | / |
| | | | IVF | 11 | 8 | unknown | unknown | 0 | / | / |
| 6 | 44 | 15 | ICSI | 13 | unknown | 4 | 4 | 0 | / | / |
| | | | ICSI | 4 | unknown | 2 | 2 | 2 | / | Failure |
| | | | ICSI | 8 | unknown | 6 | unknown | 0 | / | / |
| 7 | 35 | 8 | ICSI | 18 | 17 | 7 | 7 | 0 | 2 | Failure |
| | | | ICSI | 9 | 6 | 6 | 5 | 2 | / | Embryo arrest at 12 weeks |
| | | | ICSI | 6 | 6 | 6 | 5 | 2 | / | Failure |
| | | | ICSI | 12 | 7 | 7 | 7 | 1 | 0 | Failure |
| 8 | 29 | 3 | IVF | 5 | 5 | 3 | 3 | 0 | / | / |
| | | | ICSI | 10 | 10 | 8 | 8 | 2 | / | Failure |
| 9 | 28 | 5 | IVF | 20 | 20 | 14 | 14 | 2 | 0 | / |
| 10 | 36 | 7 | IVF | 7 | unknown | unknown | unknown | 0 | 0 | / |
| | | | ICSI | 2 | 2 | 2 | 2 | 0 | 0 | / |

/ refers to not applicable

**Supplemental Table 3. Results of NCBI/Blastp**

| Description | Sequence ID |
|---|---|
| Transcription initiation factor TFIID subunit 1 | XP_047298347.1 |
| Transcription initiation factor TFIID subunit 1-like | NP_722516.1 |
| KIAA2022 | EAW98638.1 |
| TAF4 RNA polymerase II | NP_003176.2 |
| TBP-associated factor 1 isoform 2 variant | BAD92553.1 |
| FACT complex subunit SPT16 | NP_009123.1 |
| Human TFIID bound to promoter DNA and TFIIA | 6MZM_D |
| TBP-associated factor | AAC50901.1 |
| Dystonia 3 (with Parkinsonism) | CAM98556.1 |
| RSL1D1 protein | AAI07783.1 |
| SURF6 protein | AAH03001.1 |
| Unnamed protein product | BAG51440.1 |
| Structure of the histone chaperone CIA/ASF1-double bromodomain complex linking histone modifications and site-specific histone eviction | 3AAD_A |
| Myosin phosphatase Rho-interacting protein | XP_011522064.1 |
| Rho-interacting protein 3 | BAC78198.1 |
| MPRIP protein | AAH09982.2 |
| N-acetyltransferase ESCO1 | NP_443143.2 |
| Establishment of cohesion 1 homolog 1 | AAH89426.1 |
| Intermediate filament family orphan 2 | NP_001129737.1 |
| Probable RNA-binding protein 19 | NP_001140170.1 |
| Unnamed protein product | BAB14757.1 |

| | |
|---|---|
| Splicing factor YJU2 | NP_060544.2 |
| Crystal Structure of Ubl123 with an EZH2 peptide | 6P5L_D |
| Biorientation of chromosomes in cell division protein 1-like 1 isoform X1 | XP_011512129.1 |
| Zinc finger protein 40 | NP_002105.3 |
| Human immunodeficiency virus type I enhancer binding protein 1 | EAW55310.1 |

**Supplemental Table 4. In vitro development of *Kpna2*$^{-/-}$ embryos (n = 6)**

| | | Incubation | | | |
|---|---|---|---|---|---|
| | | 24 h | 48 h | 72 h | 108 h |
| WT | Fragmented/degenerated | 0 | 0 | 5 | 23 |
| | One-cell | 2 | 2 | 1 | 1 |
| | Two-cell | 149 | 12 | 9 | 6 |
| | Four-cell | | 137 | 0 | 0 |
| | Morula | | | 136 | 2 |
| | Blastocyst | | | | 119 |
| *Kpna2*$^{-/-}$ | Fragmented/degenerated | 8 | 3 | 15 | 82 |
| | One-cell | 19 | 10 | 9 | 5 |
| | Two-cell | 128 | 142 | 102 | 62 |
| | Four-cell | 1 | 1 | 30 | 7 |
| | Morula | | | 0 | 0 |
| | Blastocyst | | | | 0 |

**Supplemental Table 5. In vitro development of *Rsl1d1*$^{OO-/-}$ embryos (n = 4)**

| | | Incubation | | | |
|---|---|---|---|---|---|
| | | 24 h | 48 h | 72 h | 108 h |
| WT | Fragmented/degenerated | 0 | 0 | 0 | 14 |
| | One-cell | 0 | 0 | 0 | 0 |
| | Two-cell | 72 | 4 | 0 | 0 |
| | Four-cell | | 68 | 4 | 0 |
| | Morula | | | 68 | 0 |
| | Blastocyst | | | | 58 |
| *Rsl1d1*$^{OO-/-}$ | Fragmented/degenerated | 2 | 9 | 19 | 23 |
| | One-cell | 20 | 15 | 8 | 7 |
| | Two-cell | 40 | 37 | 33 | 30 |
| | Four-cell | | 1 | 2 | 2 |
| | Morula | | | 0 | 0 |
| | Blastocyst | | | | 0 |

**Supplemental Table 6. Primers used for plasmid construction and real-time PCR**

| Primer name | Sequence (5'-3') | Application |
|---|---|---|
| PCMV6-Flag-KPNA7-F | GAGGCGATCGCATGGATTACAAGGATGACGACGATAAGCCGACCTT AGATGCTCCAGAA | PCR for plasmid construction |
| PCMV6-Flag-KPNA7-R | GCGACGCGTCTATTTTTTGCTAAGCATTCATAATCTATAAATTCATA ATCTTGGTCT | |
| PCMV6-Flag-mKpna7-F | GAGGCGATCGCATGGATTACAAGGATGACGACGATAAGGCTACCTC AAAGGCTCCCAAA | |
| PCMV6-Flag-mKpna7-R | GCGACGCGTTCACACTCTCAGCCCAGGCCCGGGC | |
| hKPNA7-203-F | TTCCTAGCCTTGATTTCACCCACCCTGCCG | |
| hKPNA7-203-R | ATGTGGGATGGCATTGCTTGTGATGACGTT | |
| hKPNA7-212-F | TGATCACATTTCTGCGGAACATCACGTGGA | |
| hKPNA7-212-R | GCAGGGTGGGTGAAATCAAGGCTAGGAGAT | |
| hKPNA7-175F | AAGGCAGTGTGGGCTCTTGGTAA | |
| hKPNA7-175-R | TTCACACACAGCCACGTTGGAGG | |
| hKPNA7-152-F | ATGGTAGAAGGGGGAGCCATCC | |
| hKPNA7-152-R | GGCACGAGTCTGCTCCGAAG | |
| hKPNA7-451-F | CTGATAGAAGAACTTGGTGGGATCGAT | |
| hKPNA7-451-R | AGGTTTTCCTTCTCAGACCGTTTCT | |
| mKpna7-222-F | TTCATCAACCTTATTTCAAAAGGCATACCAATC | |
| mKpna7-222-R | GTGTGGGATGGCATTATTGGAGATGAC | |
| mKpna7-231-F | TAATCACATTTCTTCGGAACATCTCATGGAC | |
| mKpna7-231-R | GTATGCCTTTTGAAATAAGGTTGATCAGGTGTGG | |

| | | |
|---|---|---|
| mKpna7-173-F | AAGGCAGTGTGGGCCCTTGGGAATA | |
| mKpna7-173-R | CTCAGACACTGTCAGGTGTGGGGAA | |
| mKpna7-150-F | ATGGTGAAAGAGGGTGCCATTCAG | |
| mKpna7-150-R | AGCTCGGGTCTGCTCTGAAG | |
| PCMV6-hKPNA2-Ha-F | GAGGCGATCGCATGTCCACCAACGAGAATGCTAAT | |
| PCMV6-hKPNA2-Ha-R | GCGACGCGTAAAGTTAAAGGTCCCAGGAGCCCCAT | |
| PCMV6-mKpna2-Ha-F | GAGGCGATCGCATGTCCACGAACGAGAATGCTAACTTACC | |
| PCMV6-mKpna2-Ha-R | GCGACGCGTGAAGTTAAAGGTCCCAGGAGCTCCAT | |
| pGEX4T-mRSL1D1-GFP-F | CGCGTGGATCCCCGGAATTCATGGAGGATTCGGCCTCGGC | |
| RSL1D1-GFP-R | AGTTCTTCTCCTTTACTGGTCGACTGGGGTACTTTGGG | |
| RSL1D1-GFP-F | CCCCAGTCGACCAGTAAAGGAGAAGAACTTTTCACTGGAGTTG | |
| pGEX4T-mRSL1D1-GFP-R | GTCAGTCACGATGCGGCCGCCTATTTGTATAGTTCATCCATGCCATG TGTAATCCC | |
| PCMV6-hRSL1D1-Ha-F | AGATCTGCCGCCGCGATCGCATGGAGGATTCGGCCTCGG | |
| PCMV6-hRSL1D1-Ha-R | TCGAGCGGCCGCGTACGCGTGGTCGACTGGGGTACTTTGGG | |
| PCMV6-mRSL1D1-Ha-F | GATCTGCCGCCGCGATCGCATGAAGGGCTCTGCGTCCG | |
| PCMV6-mRSL1D1-Ha-R | TCGAGCGGCCGCGTACGCGTGTTTGAGTGGGCCGCCTTG | |
| mRSL1D1-sysnmut-F | GTCTGCTTATTCACAAAAGATGAATTCGATTCACC | |
| mRSL1D1-sysnmut-R | GTCGGATGATTCGGAGAGAATGCTATGAGGCAAAG | |
| pCR3.1-mClover-mRSL1D1-F | CTTCAGATCTGGTTACGCGTATGAAGGGCTCTGCGTCCGAATC | |
| pCR3.1-mClover-mRSD1-R | TTCTGGATATACCACAGCGATCGCCTAGTTTGAGTGGGCCGCCTTG | |
| KPNA7-mRT-F | GACATCATTTCTTATCTCCTCCAG | For real-time quantitative PCRs |
| KPNA7-mRT-R | GCAAACTGTCACCATCTCCATC | |
| KPNA6-mRT-F | CACTGCTGGCAACAGGGCTCAA | |

| | |
|---|---|
| KPNA6-mRT-R | GCAGCCCAGTGATACCAGGTAC |
| KPNA4-mRT-F | CAATCTCATAGAAGAATGTGGTG |
| KPNA4-mRT-R | CTCTGTTGGTACATTGGTCGAT |
| KPNA3-mRT-F | TGAGATCATAGAAGAGTGTGGAG |
| KPNA3-mRT-R | TGAAGGTTGGCTGTTGGGTC |
| KPNA2-mRT-F | TGATGCTACTTCTCCGCTACAG |
| KPNA2-mRT-R | GGATGATGTTGTCTATAGGAGG |
| KPNA1-mRT-F | ACAGCTGGAAATAGGGCACAGA |
| KPNA1-mRT-R | GATACAACCCAGTTCTACTAGGTAC |
| KPNA7-hRT-F | GTGATGGCCCAGAGTTCAGAGATAA |
| KPNA7-hRT-R | CATCCGAGAGAACCTCACTGTCCTG |
| KPNA6-hRT-F | GCATTCAGCTCCGGAAGCAGAA |
| KPNA6-hRT-R | GTGTGGTTGCTAACTGCAGGT |
| KPNA5-hRT-F | TAGTTCCACTGTACCCATTCCAGAG |
| KPNA5-hRT-R | TCTCTGTACAACTCCTGGTTTCTGT |
| KPNA4-hRT-F | ACTATGAGAAGACAACGAAATGAAGTTG |
| KPNA4-hRT-R | TAGCAGCTTGAACTGCACTTAATTG |
| KPNA3-hRT-F | TGAAGTGACAGTGGAACTGCGGAAG |
| KPNA3-hRT-R | CTTGCTGCCTGGACAGCACTCAATT |
| KPNA2-hRT-F | GAAATGAGGCGTCGCAGAATAGAGG |
| KPNA2-hRT-R | CTGGCAGCTTGAGTAGCTTGGAG |
| KPNA1-hRT-F | CAGATTAGTAACATGGAGATGGCACCA |
| KPNA1-hRT-R | CCACAAACCTGGCCACTACTCCT |
| GAPDH-hRT-F | GGAGCGAGATCCCTCCAAAAT |

| | |
|---|---|
| GAPDH-hRT-R | GGCTGTTGTCATACTTCTCATGG |
| ACTB-hRT-F | ATGATGATATCGCCGCGCTC |
| ACTB-hRT-R | AATCCTTCTGACCCATGCCC |
| Kpna2-mRT-F | TGATGCTACTTCTCCGCTACAG |
| Kpna2-mRT-R | GGATGATGTTGTCTATAGGAGG |
| Kpna7-mRT-F | GACATCATTTCTTATCTCCTCCAG |
| Kpna7-mRT-R | GCAAACTGTCACCATCTCCATC |
| Actb-mRT-F | CAGCTTCTTTGCAGCTCCTT |
| Actb-mRT-R | AGTCCTTCTGACCCATTCCCA |
| Bclaf1-mRT-F | ATGGCACCTGTTCCTCTTGACG |
| Bclaf1-mRT-R | GAGGCAGCTTAATGTGGTCAAAG |
| Crcp-mRT-F | CAAAGAGCAGCGGAAGGAGAGT |
| Crcp-mRT-R | GACAATCGCTGGACTCTGGTTC |
| Polr3k-mRT-F | AGAAGTGGACGACGTGCTTGGT |
| Polr3k-mRT-R | AGAAGGTGGTCATTGGCTCGTC |
| Znrd1-mRT-F | GTCTGTGGATGAGGGACCTGAA |
| Znrd1-mRT-R | AGACCGTCTGTCCTTCATCAGC |
| Zmpste24-mRT-F | CTACTCAGAGGTGGAAGGCACT |
| Zmpste24-mRT-R | ACAGCAGGAACACCAATGACTGA |
| Xrn2-mRT-F | CAGTTTGGACACGAGGTCAAGG |
| Xrn2-mRT-R | ACTCTCGAAGGACATTCAGCCG |
| Gtf2f2-mRT-F | ACAGTGTTTACGGAGAGCTCCTCAG |
| Gtf2f2-mRT-R | CAGCTGTTGTGAGAGCCTTACAGGT |
| Wdr61-mRT-F | GCTCCTTGTCACGGCTTCAGAT |

| | |
|---|---|
| Wdr61-mRT-R | GTGTCATCAGGACAGAACGCAAC |
| Dhx36-mRT-F | GTCTTTCTACCAGGCTGGGACA |
| Dhx36-mRT-R | GTGTCTGGTTGACGGTAGGCAT |
| Aars-mRT-F | CCAATCAGACTCCAGTGGTAGC |
| Aars-mRT-R | GCTCCGCATAGAAACAGGTCTTGT |

Abbreviations: F forward, R reverse
